# RUNX1B Expression Is Highly Heterogeneous and Distinguishes Megakaryocytic and Erythroid Lineage Fate in Adult Mouse Hematopoiesis

**DOI:** 10.1371/journal.pgen.1005814

**Published:** 2016-01-25

**Authors:** Julia E. Draper, Patrycja Sroczynska, Olga Tsoulaki, Hui Sun Leong, Muhammad Z. H. Fadlullah, Crispin Miller, Valerie Kouskoff, Georges Lacaud

**Affiliations:** 1 Cancer Research UK Stem Cell Biology Group, Cancer Research UK Manchester Institute, The University of Manchester, Manchester, United Kingdom; 2 Biotech Research and Innovation Centre, University of Copenhagen, Copenhagen, Denmark; 3 Centre for Epigenetics, University of Copenhagen, Copenhagen, Denmark; 4 Cancer Research UK Applied Computational Biology and Bioinformatics Group, Cancer Research UK Manchester Institute, The University of Manchester, Manchester, United Kingdom; 5 Cancer Research UK Stem Cell Haematopoiesis Group, Cancer Research UK Manchester Institute, The University of Manchester, Manchester, United Kingdom; Cincinnati Children's Hospital Medical Center, UNITED STATES

## Abstract

The Core Binding Factor (CBF) protein RUNX1 is a master regulator of definitive hematopoiesis, crucial for hematopoietic stem cell (HSC) emergence during ontogeny. RUNX1 also plays vital roles in adult mice, in regulating the correct specification of numerous blood lineages. Akin to the other mammalian *Runx* genes, *Runx1* has two promoters *P1* (distal) and *P2* (proximal) which generate distinct protein isoforms. The activities and specific relevance of these two promoters in adult hematopoiesis remain to be fully elucidated. Utilizing a dual reporter mouse model we demonstrate that the distal *P1* promoter is broadly active in adult hematopoietic stem and progenitor cell (HSPC) populations. By contrast the activity of the proximal *P2* promoter is more restricted and its upregulation, in both the immature Lineage^-^ Sca1^high^ cKit^high^ (LSK) and bipotential Pre-Megakaryocytic/Erythroid Progenitor (PreMegE) populations, coincides with a loss of erythroid (Ery) specification. Accordingly the PreMegE population can be prospectively separated into “pro-erythroid” and “pro-megakaryocyte” populations based on *Runx1 P2* activity. Comparative gene expression analyses between *Runx1 P2*^*+*^ and *P2*^*-*^ populations indicated that levels of CD34 expression could substitute for *P2* activity to distinguish these two cell populations in wild type (WT) bone marrow (BM). Prospective isolation of these two populations will enable the further investigation of molecular mechanisms involved in megakaryocytic/erythroid (Mk/Ery) cell fate decisions. Having characterized the extensive activity of *P1*, we utilized a *P1-GFP* homozygous mouse model to analyze the impact of the complete absence of *Runx1 P1* expression in adult mice and observed strong defects in the T cell lineage. Finally, we investigated how the leukemic fusion protein *AML1-ETO9a* might influence *Runx1* promoter usage. Short-term AML1-ETO9a induction in BM resulted in preferential *P2* upregulation, suggesting its expression may be important to establish a pre-leukemic environment.

## Author Summary

The transcription factor RUNX1 is considered a master regulator of adult and embryonic blood cell production. Mutations in *RUNX1* cause defects in different blood lineages in human patients and mouse models, including leukemia and blood clotting defects due to a shortage of platelet-producing megakaryocytes. Together with the other *RUNX* genes present in mammals, *RUNX1* is expressed from two promoters, which produce several distinct RNA transcripts and protein isoforms. To investigate the timing and localization of the expression of these two promoters (termed distal and proximal), we created a mouse model with reporter genes expressed under the control of the *Runx1* promoters. We previously described the activities of the *Runx1* promoters at the initiation of blood production in the developing embryo. We now investigate the output from the two promoters in adult organs, including bone marrow, spleen and thymus. We show here that the distal *Runx1* promoter is highly expressed but the proximal promoter is more restricted and in particular marks the point in adult blood production where the red blood cell and megakaryocyte pathways separate. The different proteins produced by these two *Runx1* promoters may therefore have different roles in driving the production of these two distinct cell types.

## Introduction

Adult hematopoiesis is orchestrated by a series of lineage fate decisions that control the specification of mature erythroid, myeloid and lymphoid blood cells from pluripotent HSCs. RUNX transcription factors play key roles at different stages, activating or repressing transcriptional targets through DNA binding in association with other lineage-specific and ubiquitous transcription factors and cofactors [1,2]. RUNX1 (also known as Acute Myeloid Leukemia 1 or AML1) is a master regulator of definitive hematopoiesis, broadly expressed in HSCs, progenitors and mature populations, with the exception of terminally differentiated erythrocytes [3–5]. RUNX1 activity is vital for the embryonic establishment of normal adult hematopoiesis through the regulation of HSPC emergence in a process termed endothelial-to-hematopoietic transition (EHT) [6–12]. Conditional deletion of *Runx1* in adult mice, meanwhile, results in hematological imbalances such as decrease of peripheral blood lymphocytes, expansion of monocytes and granulocytes and impaired T cell maturation [13–15]. RUNX1 is also critical in megakaryocytic maturation and platelet production [16,17]. The requirement for RUNX1 in adult HSC maintenance is more controversial, with assertions of impaired long-term repopulating ability in *Runx1*-null HSCs due to increased stem cell exhaustion being increasingly challenged [6,18,19].

The importance of normal CBF function extends to malignant hematopoiesis, with *RUNX1* or *CBFB* mutations found in over 20% of acute myeloid and lymphoid leukemia cases [20]. Although impaired RUNX1 activity is frequently important for establishing a pre-leukemic stage, WT RUNX1 protein is nonetheless necessary for maintaining AML1-ETO Acute Myeloid Leukemia (AML) [21,22]. Consequently, the investigation of RUNX1’s expression and function in hematopoiesis is of considerable interest to developmental biologists and clinical researchers alike.

All vertebrate *Runx* genes contain two alternative promoters, a *distal P1* promoter and a *proximal P2* promoter thought to represent the initial “primitive” promoter [23–25]. The major protein isoforms produced from the *P1* and *P2* promoters, RUNX1C and RUNX1B respectively, differ in their N-terminal amino acid sequences; RUNX1C is 14 amino acids longer and begins with the MASDS sequence whereas RUNX1B begins with MRIPV, a feature conserved in mice and humans [26,27]. *P2* is the more active promoter at the onset of definitive hematopoiesis in the E7.5 embryo [28,29]. *P1* activity is subsequently upregulated, enriched in definitive hematopoietic culture colony-forming unit (CFU-C) populations from E8.5 onwards [29]. Analyses on whole cell populations revealed a remarkable switch to *P1*-dominant *Runx1* expression at the fetal liver stage that is maintained in adult BM populations [28,29]. At this stage *P2* activity is only detected in some specific adult hematopoietic subsets. However, the exact cell populations defined by the activities of *P1* and *P2* remain largely unknown.

To define the activities of the *Runx1* promoters in adult HSPCs we utilized a previously described *distal-*Green Fluorescent Protein *(GFP)*, *proximal-*truncated human *CD4 (hCD4)* (*P1-GFP*::*P2-hCD4*) dual reporter knock-in mouse line [29]. We observed that all *Runx1*‐positive adult BM populations expressed *P1‐GFP*, whereas *P2‐hCD4* expression was highly restricted. Phenotypic HSCs expressed solely *P1‐GFP*, with upregulation of *P2‐hCD4* in CD48‐positive multipotent progenitors (MPPs) coinciding with a significant downregulation of erythroid output. We also found that the PreMegE population could be prospectively separated into *P2‐hCD4*^‐^ “pro‐erythroid” and *P2‐hCD4*^+^ “pro‐megakaryocyte” populations. Global gene expression analyses identified various candidate cell surface markers which were differentially expressed between the two PreMegE subpopulations. Among them, differential expression of the hematopoietic cell antigen CD34 enabled the prospective isolation of CD34^-^ “pro‐erythroid” and CD34^+^ “pro‐megakaryocyte” PreMegEs from WT BM.

To further investigate the potential functional significance of the dominance of RUNX1C in adult hematopoiesis, we investigated the impact of its absence in adult mice and found it to recapitulate certain phenotypes observed in complete *Runx1* knockout mouse models. We observed perturbations in platelet versus erythroid output and altered splenic CD4 SP and CD8 SP specification, suggesting certain lineages were more dependent on specific RUNX1C-associated activity than others. Finally, we probed the potential specific RUNX1 isoform requirements in AML by analyzing the impact of AML1-ETO oncogene expression on *Runx1* promoter usage. Interestingly, AML1-ETO expression appeared to promote *Runx1 P2* over *P1* expression in several HSPC populations, suggesting that the *Runx1* isoforms may have specific functions both in normal and malignant hematopoiesis.

## Results

### *Runx1 P1* is the dominant promoter in adult hematopoiesis

**Fig 1:**
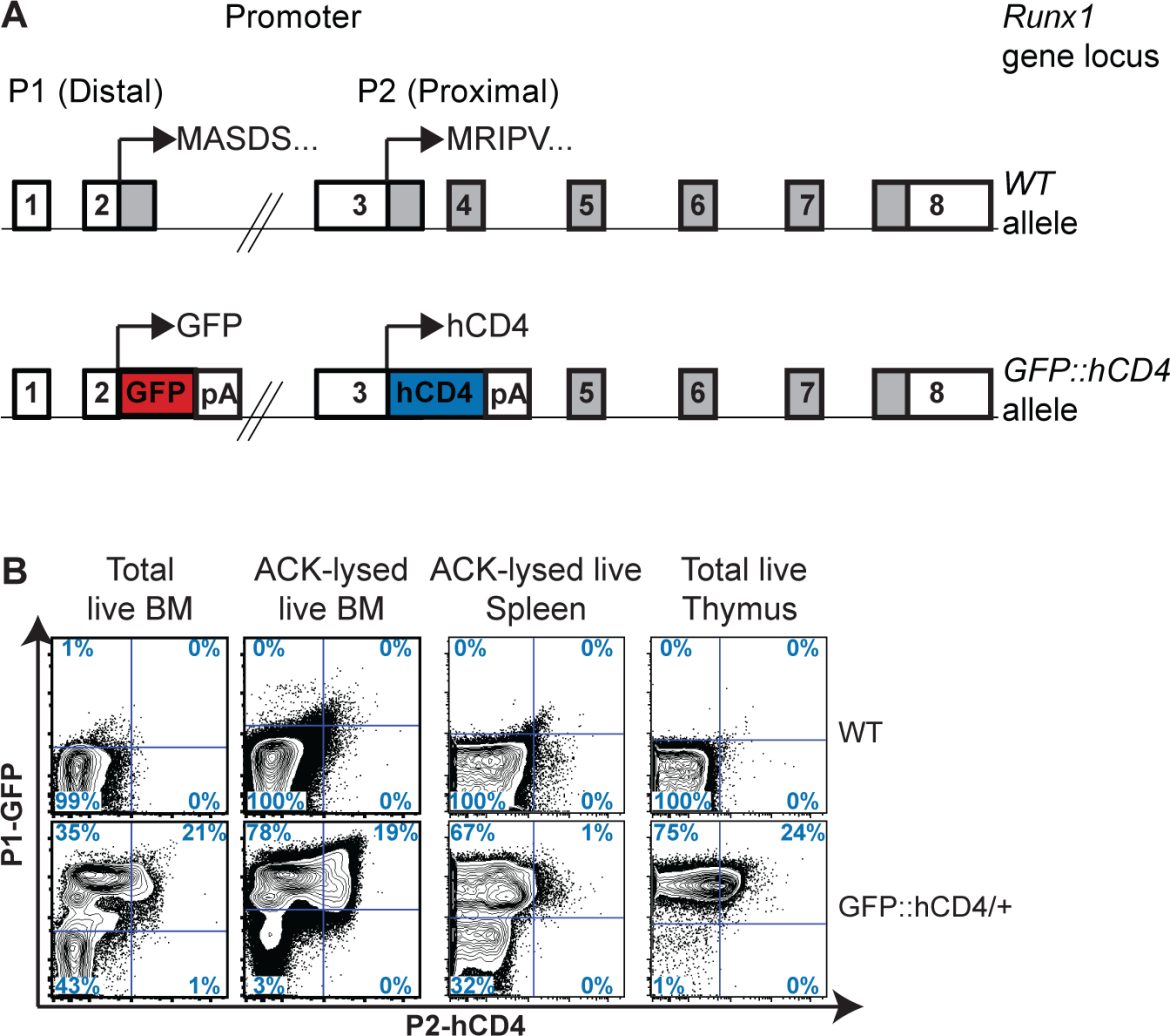
*Runx1* promoter *P1* and *P2* expression in adult hematopoietic organs. (A) Schematic diagrams of the *Runx1 WT* (top) and *P1-GFP*::*P2-hCD4* dual reporter (*GFP*::*hCD4*, bottom) alleles. Expression of GFP is directed by *Runx1* promoter *P1* and a truncated hCD4 reporter is expressed under the control of *Runx1* promoter *P2*. (B) Contour plots of *Runx1 P1-GFP* and *P2-hCD4* expression in unfractionated total (far left panel) and ACK buffer lysed (middle left panel) adult BM, spleen (middle right panel) and thymus (far right panel) in *WT* (top) and *P1-GFP*::*P2-hCD4/+* (bottom) mice. Representative data of three independent experiments are shown.

Utilizing the *P1-GFP*::*P2-hCD4* reporter mouse model [29], we traced *Runx1* expression for both promoters *in vivo* at a single cell level in adult mice (with flow cytometry gates based on the WT control tissues) (Fig 1A and 1B). We observed substantial heterogeneity of *Runx1* expression within adult BM; approximately 55% of all BM cells were *P1-GFP* positive, almost 21% co-expressing *P2-hCD4* (Fig 1B). Red blood cell lysis (using Ammonium-Chloride-Potassium (ACK) buffer) led to the depletion of *P1-GFP*^*-*^ *P2-hCD4*^*-*^ cells; 97% of remaining cells expressed *P1-GFP* with 20% co-expressing *P2-hCD4*. No *P1-GFP*^*-*^ *P2-hCD4*^*+*^ cells were observed. In the spleen, approximately 70% of cells expressed *P1-GFP* but only 1% co-expressed *P2-hCD4*, whereas in the thymus almost 100% of cells expressed *P1-GFP*, a quarter of which also expressed *P2-hCD4*. Altogether these results establish, in line with other reports, that *P1* is the dominant *Runx1* promoter in adult hematopoietic populations and that the activity of P2 is much more restricted [28].

**Fig 2:**
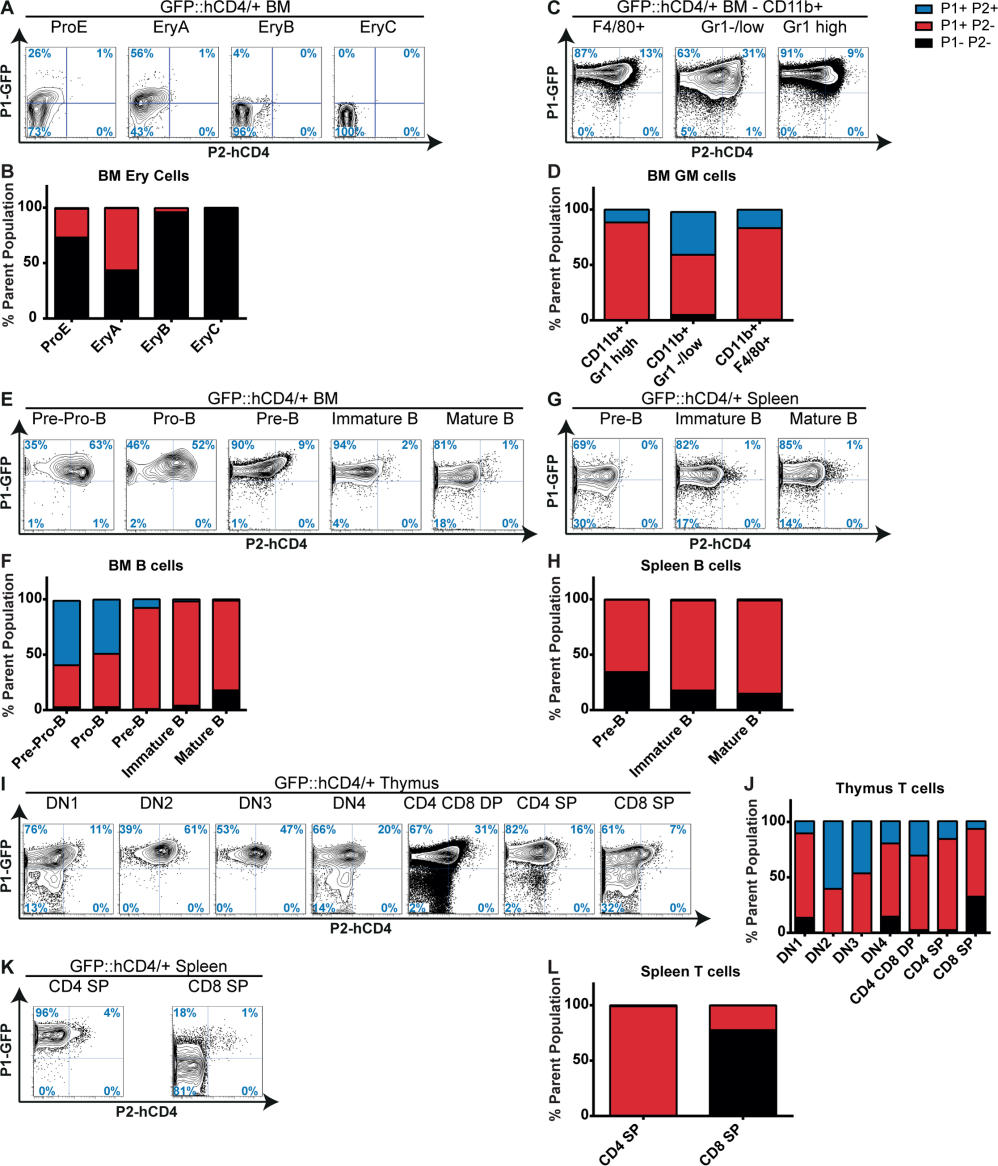
*Runx1 P1* and *P2* expression in mature hematopoietic lineages. (A, C, E, G, I, K) Contour plots of *Runx1 P1-GFP* and *P2-hCD4* expression in BM erythroid (A), granulocytic/macrophage (C) and B lymphocyte populations (E), spleen B (G) or T (K) lymphocytes and thymocytes (I), as defined in S1 Fig. (B, D, F, H, J, L) Numbers of *Runx1 P1*^*-*^ *P2*^*-*^, *P1*^*+*^ *P2*^*-*^ and *P1*^*+*^ *P2*^*+*^ cells as a proportion of defined BM erythroid (B), granulocytic/macrophage (D) and B lymphocyte populations (F), spleen B (H) or T (L) lymphocytes and thymocytes (J). Representative data of three independent experiments are shown.

Taking advantage of our reporter model, we pursued a detailed examination of *P1-GFP* and *P2-hCD4* expression in mature lymphoid and erythro-myeloid populations (Figs 2 and S1).

*Runx1* is expressed in definitive erythroid precursors, where it is involved in the regulation of erythroid gene expression as part of a core transcription factor complex, but is subsequently downregulated in mature erythrocytes [3–5,28,30]. Correspondingly, *P1-GFP* expression was restricted to 26% of the proerythroblast (CD71^high^ Ter119^int^, ProE), 56% of the basophilic erythroblast (CD71^high^ Ter119^high^ FSC^high^, EryA) and 4% of the late basophilic/polychromatic erythroblast (CD71^high^ Ter119^high^ FSC^low^, EryB) fractions whilst being completely absent in the most mature CD71^low^ Ter119^high^ FSC^low^ (orthochromatic erythroblasts, reticulocytes, red blood cells, EryC) compartment (Figs 2A, 2B, and S1A). *P2-hCD4* was expressed in less than 1% of Ter119^+^ erythroid cells, being apparently entirely dispensable for adult erythropoiesis. The low level of expression from both *Runx1 P1* and *P2* promoters, particularly the latter, in WT erythroid lineage cells was confirmed at the RNA level by qPCR (S2A Fig).

By contrast to the restricted expression observed in the erythroid lineage, *Runx1 P1-*GFP was expressed in almost 100% of mature myeloid CD11b^+^ BM cells (Figs 2C, 2D and S1B). Of these, *P2-hCD4* was co-expressed in 12% of Gr1^high^ granulocytes, 39% of Gr1^-/low^ monocytic/immature granulocyte cells and 17% of F4/80^+^ macrophages. The decreased *P2* activity in the more mature granulocytic/macrophage (GM) fractions suggests a diminished role for RUNX1B as myeloid differentiation progresses. This also appears to be the case for terminal lymphoid differentiation, as *P2-hCD4* co-expression with *P1-*GFP was restricted in the B-cell lineage to 58% of the BM Pre-pro-B, almost half (49%) of the Pro-B and just 8% of the Pre-B progenitors (Figs 2E–2H, S1C and S1D). *P1-GFP* was expressed in over 90% of BM B cell progenitors but was reduced to approximately 80% of mature BM and spleen B cells. Finally, thymic T cells were highly enriched in the *P1-GFP*^*+*^ *P2-hCD4*^*-*^ fraction but *P2-hCD4* activity appeared to peak in the CD4 CD8 double negative 2 (DN2) fraction at approximately 61% (Figs 2I, 2J and S1E). Interestingly, the more mature spleen CD4 and CD8 single positive (SP) T cell subsets displayed greater heterogeneity than their thymic counterparts; almost 100% of CD4 SP cells express *P1-GFP* whilst this is the case for only 20% of CD8 SP cells (Figs 2K, 2L and S1F). Relative quantitation of the *Runx1* isoforms’ expression revealed comparatively high *P1* and *P2* activity in the GM, B and T lineages, peaking in the early thymic T cell CD4 CD8 DN population and provides direct evidence that the *P1-GFP*::*P2-hCD4* reporters faithfully represent *WT Runx1* expression throughout adult hematopoiesis (S2A–S2C Fig). Overall, *P1* clearly dominates, accounting for over 80% of *Runx1* expression in all analyzed lineage positive populations. Nonetheless, strong *P2* expression was observed in CD11b^+^ GR1^+^ GM cells, Pre-pro/pro/pre-B cells and CD4 CD8 DN T cells, decreasing substantially in the more mature IgM^+^ B and CD4/CD8^+^ T cells. These results indicate that *P1* is the dominant *Runx1* promoter in terminally differentiated hematopoietic cells and suggest that downregulation of *P2* is required for maturation to occur. We therefore decided to determine whether *P2* expression has a greater prominence and significance in immature HSPC subsets.

### Upregulation of *Runx1 P2* in HSPCs marks a loss of erythroid potential

**Fig 3:**
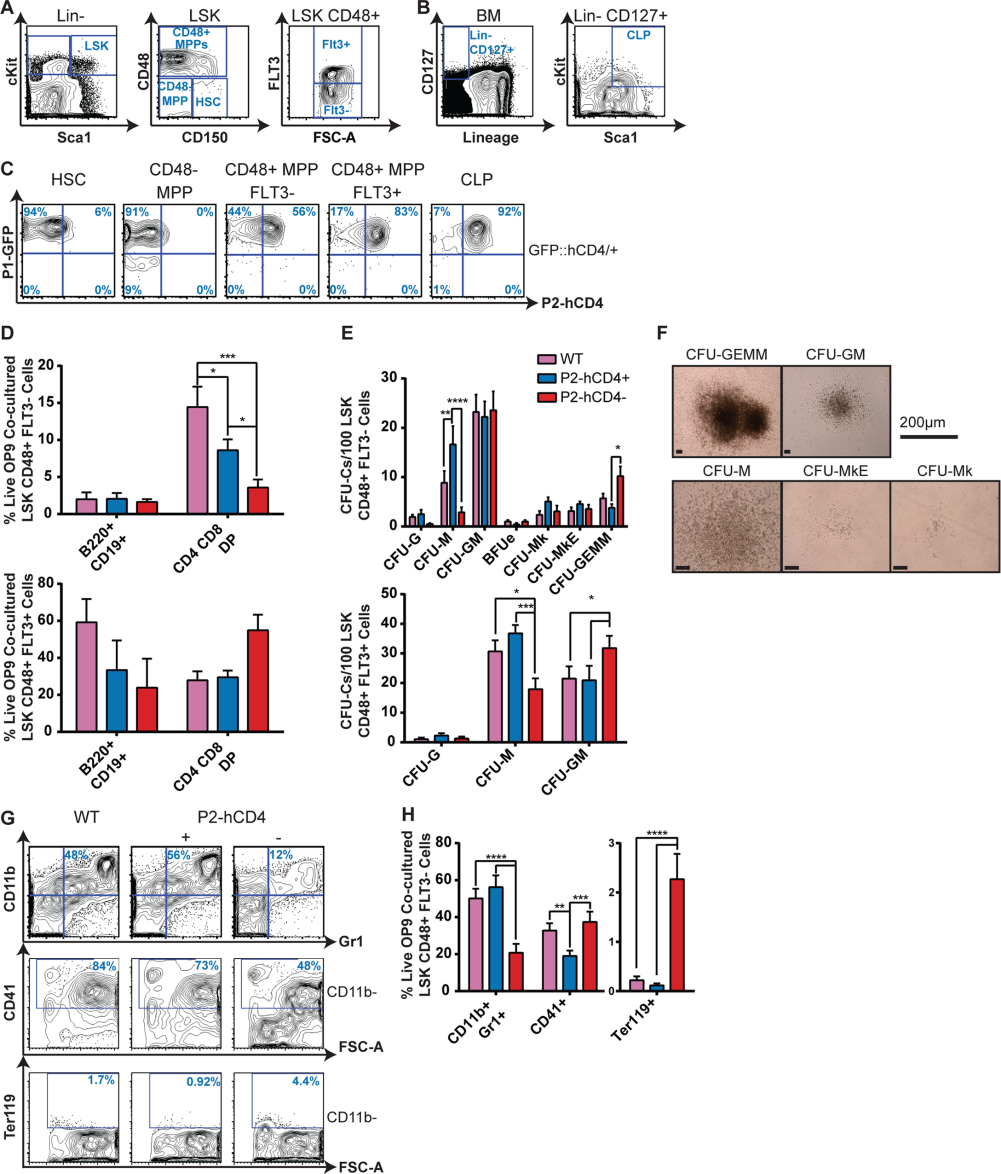
Upregulation of *Runx1 P2* in HSPCs marks a loss of erythroid potential. (A–B) Contour plots showing immature adult BM Lineage (Lin) negative hematopoietic progenitors. (A) The LSK fraction can be separated into distinct HSCs, CD48^-^ MPPs and more mature CD48^+^ MPPs on the basis of CD150, CD48 and FLT3 expression. (B) The CLP is characterized as Lin^-^ CD127^+^ cKit^low^ Sca1^low^. (C) Representative FACS plots of P1-GFP/P2-hCD4 expression in HSCs, CD48^-^ MPPs, CD48^+^ FLT3^-^ (LSK48F^-^) MPPs, CD48^+^ FLT3^+^ (LSK48F^+^) MPPs and CLPs. (D) Numbers of B220^+^ CD19^+^ B cells and CD4^+^ CD8^+^ DP T cells produced following co-culture of *WT*, *P1*^*+*^ *P2*^*-*^ and *P1*^*+*^ *P2*^*+*^ LSK48F^-^ MPPs (top) and LSK48F^+^ MPPs (bottom) with OP9 (B cells) or OP9-DL1 (T cells) for 21 days. (n = 4). (E) CFU-C activity of *WT*, *P1*^*+*^ *P2*^*-*^ and *P1*^*+*^ *P2*^*+*^ LSK48F^-^ MPPs (top) and LSK48F^+^ MPPs (bottom) following culture in pro-myeloid semi-solid methylcellulose-based medium. (LSK48F^-^, n = 7; LSK48F^+^, n = 6.) (F) Photographs of representative LSK48F^-^ derived methylcellulose colonies. (G) Representative CD11b/Gr1, CD41 and Ter119 FACS plots of OP9 co-cultured LSK48F^-^ MPPs isolated on day 8. (H) Quantification of CD11b^+^ Gr1^+^ granulocyte/macrophage (GM) cells, CD41^+^ megakaryocytes and Ter119^+^ erythrocytes in the progenitor/OP9 co-culture assays (n = 5).

To examine the relative activities of the two *Runx1* promoters in the most immature hematopoietic compartments, we separated the LSK fraction into phenotypic HSC and MPP fractions (Fig 3A). We observed that only the *P1* promoter was active in the HSCs and CD48- MPPs (Fig 3C). The upregulation of CD48 expression coincides with the loss of long-term repopulating ability, the LSK CD48^+^ fraction consisting of a mixture of lymphoid and myeloid progenitors with varying multipotentiality. FMS-Like Tyrosine Kinase 3 (FLT3) expression marks a commitment to the GM and lymphoid lineages at the expense of Mk/Ery specification [31,32]. Increased GM/lymphoid lineage commitment appears to coincide with increased *P2* activity, as the majority of cells in the lymphoid-primed multipotent progenitor (LMPP)-enriched FLT3^+^ and the common lymphoid progenitor (CLP, Fig 3B) subsets co-expressed *P1-GFP* and *P2-hCD4* (Fig 3C). Therefore, although *P1* is the dominant *Runx1* promoter at the onset of adult hematopoiesis, our results suggest that *P2* expression imparts or at least reflects distinct lineage commitment decisions in these immature hematopoietic compartments. Consistent with this theory, *Runx1 P1* activity, as measured by quantitative RT PCR in WT BM HSPCs, peaked in the WT HSCs and decreased by approximately 50% in the FLT3^+^ MPPs, coinciding with the substantial increase in *Runx1 P2* expression (S3 Fig). The overall result is that total *Runx1* expression decreases only modestly in MPPs compared to HSCs but the relative contribution by *P1* compared to *P2* decreases substantially.

When the differences in biological potential were directly assessed in FLT3^+^ MPPs, *P2-hCD4*^*-*^ and *P2-hCD4*^*+*^ subpopulations were capable of relatively similar levels of lymphoid and myeloid differentiation (Fig 3D and 3E bottom), although the increased GM:M CFU-C ratio in the *P2-hCD4*^*-*^ subset may suggest it represents a more immature population than its *P2-hCD4*^*+*^ counterpart. However, the difference in lineage output by the FLT3^-^ subsets was more marked; *P2-hCD4*^*-*^ LSK CD48^+^ FLT3^-^ (LSK48F^-^) MPPs appeared to have reduced T cell output (Fig 3D top) but enhanced multilineage myeloid colony-forming unit potential at the expense of CFU-M output (Fig 3E and 3F). Co-culturing the LSK48F^-^ progenitors with the OP9 murine stromal cell line in myeloid differentiation media revealed that CD11b^+^/Gr1^+^ GM output was significantly decreased and CD41^+^ megakaryocytic (Mk) cell production was slightly increased in the *P2-hCD4*^*-*^ fraction as a proportion of total cells (Fig 3G and 3H). As a proportion of non-GM (CD11b^-^) cells, CD41^+^ Mk cell output was in fact significantly increased in the *P2-hCD4*^*+*^ LSK48F^-^ fraction. Most strikingly, Ter119^+^ erythroid cell output was almost entirely restricted to the *P2-hCD4*^*-*^ fraction. Our phenotypic characterization of BM HSPCs therefore demonstrate that upregulation of *Runx1 P2* not only occurs after loss of HSC activity but also coincides with a substantial decrease in erythroid specification.

To determine whether LSK48F^-^ *P2-hCD4*^*-*^ and *P2-hCD4*^*+*^ progenitors arise sequentially or independently in the hematopoietic hierarchy, sorted cells were cultured with pro-myeloid cytokines for up to 18 hours and immunophenotypically characterized (S4A and S4B Fig). Whereas the *P2-hCD4*^*+*^ fraction solely produced *P2-hCD4*^*+*^ LSK cells, *P2-hCD4*^*-*^ cultures yielded *P2-hCD4*^*-*^ and *P2-hCD4*^*+*^ LSK cells (S4B Fig). In addition, LSK48F^-^ *P2-hCD4*^*-*^ cultures produced more phenotypic erythroid (pre-erythroid colony-forming unit, PreCFUe or erythroid colony-forming unit, CFUe) or bi-potential PreMegE progenitors and fewer GM (Pre- Granulocyte-Macrophage progenitor, PreGM or Granulocyte-Macrophage Progenitor, GMP) and megakaryocyte progenitor (MkP) cells compared to LSK48F^-^ *P2-hCD4*^*+*^ cells. Altogether these data demonstrate a hierarchical relationship between an erythroid-biased *P2-hCD4*^*-*^ MPP population and increasingly pro-GM/Mk *P2-hCD4*^*+*^ progeny.

### *Runx1 P2* expression in GM-restricted progenitors enriches for monocyte/macrophage specification

**Fig 4:**
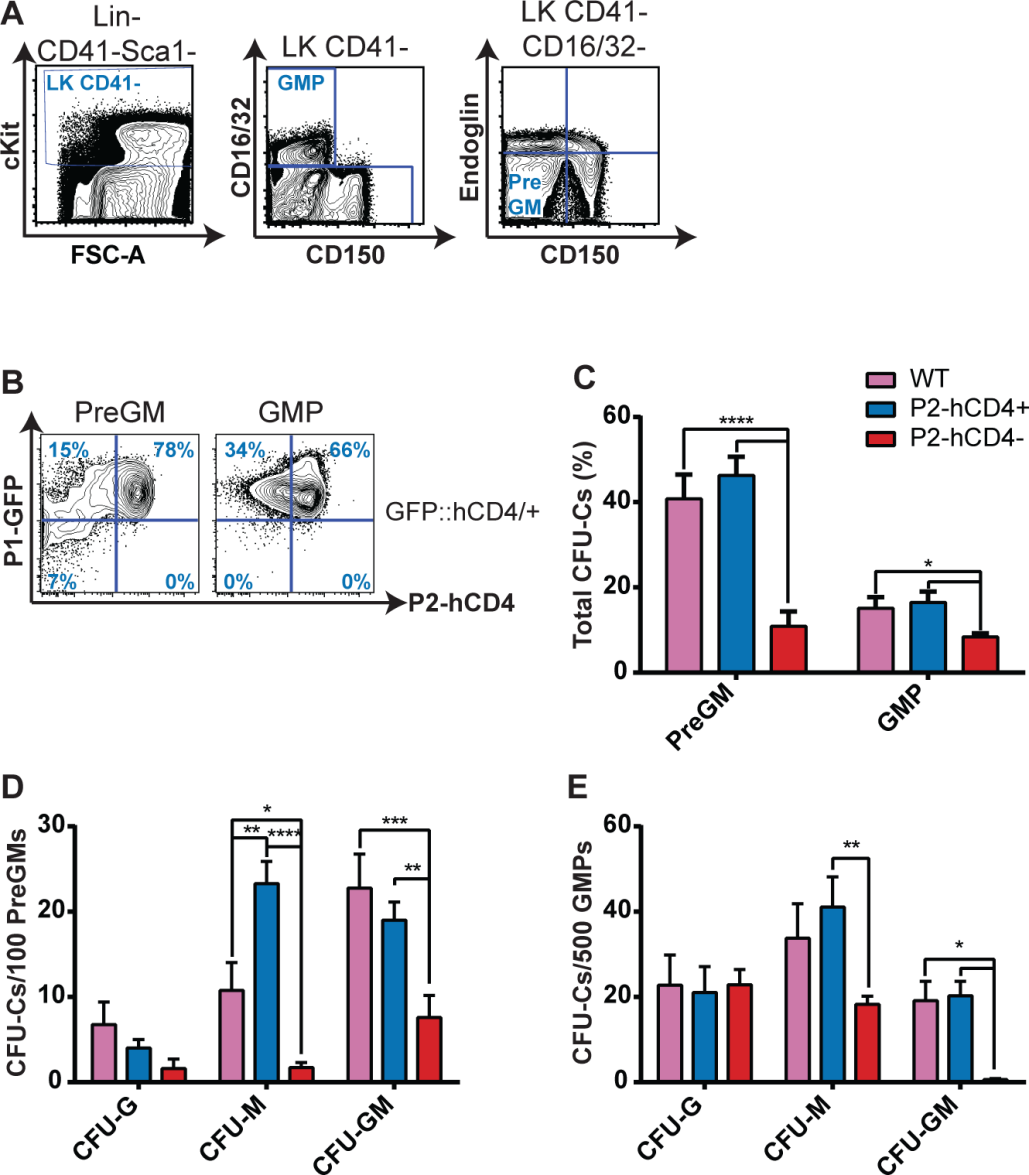
*Runx1 P2* expression in GM-restricted progenitors enriches for monocyte/macrophage specification. (A) Contour plots of adult BM Lin^-^ Sca1^-^ cKit^+^ (LK) GM progenitors. The GMP and PreGM progenitors can be distinguished on the basis of CD41, CD16/32, SLAMF1 (CD150) and Endoglin expression. (B) Representative FACS plots of P1-GFP/P2-hCD4 expression in PreGM and GMP cells. (C–E) CFU-C activity of *WT*, *P1*^*+*^ *P2*^*-*^ and *P1*^*+*^ *P2*^*+*^ PreGMs and GMPs following culture in pro-myeloid semi-solid methylcellulose-based medium. (C) Total CFU-C numbers (%). (D-E) Granulocyte (CFU-G), macrophage (CFU-M) and granulocyte/macrophage (CFU-GM) colony forming unit numbers per 100 plated PreGMs (D) or 500 plated GMPs (E). n = 4 independent experiments.

Although *Runx1 P2* expression appears to decrease as GM maturation proceeds, its expression in the earliest identified GM-restricted progenitors remained unknown. We found that approximately 80% of PreGMs and 70% of GMPs co-express *P1-GFP* and *P2-hCD4* (Fig 4A and 4B). Moreover, we found *P2* expression (as determined by qRT-PCR) to be higher in the PreGM and GMP than other analyzed WT BM HSPC populations but *P1* expression was only 50% and 25% of the level observed in HSCs (S3 Fig). High *P2* activity therefore appeared to be important for GM lineage commitment and we decided to investigate the functionality of the *P2-hCD4*^*+*^ and minority *P2-hCD4*^*-*^ GM progenitor populations. Interestingly, CFU-C activity was significantly higher in the *P2-hCD4*^*+*^ fractions, compared to the *P2-hCD4*^*-*^ populations, of both PreGM and GMP populations, reflecting higher CFU-M and CFU-GM frequencies (Fig 4C–4E). In particular, the *P2-hCD4*^*-*^ GMP fraction appeared to consist of monopotent granulocytic and monocytic/macrophage progenitors rather than bipotential GM progenitors. Liquid culture of the progenitors confirms an apparent bias against macrophage specification as F4/80^+^ cell numbers were significantly diminished in *P2-hCD4*^*-*^ PreGM and GMP cultures, whereas Gr1^high^ granulocyte output was unaltered (S5A and S5B Fig). Interestingly, production of CD11b^-^ cKit^+^ FcεR1α^+^ mast cells was also elevated in *P2-hCD4*^*-*^ cultures and more detailed analyses confirmed the absence of *P2-hCD4* expression in immunophenotypic mast cell progenitors (MCp, S5A–S5C Fig). The decreased CFU-GM activity of the *P2-hCD4*^*-*^ GM progenitors implies that they reside later in the hematopoietic hierarchy than the *P2-hCD4*^*+*^ populations, but *in vitro* lineage tracing revealed that *P2-hCD4*^*-*^ GMPs gave rise to *P2-hCD4*^*+*^ GMPs (S5E Fig). *P2-hCD4*^*-*^ PreGMs gave rise to *P2-hCD4*^*+*^ PreGMs and subsequently to *P2-hCD4*^*-*^ and *P2-hCD4*^*+*^ GMPs, the latter dominating (S5D Fig). Therefore even within the GM lineage, differential *Runx1* promoter activity appears to play a role in or at least correlate with crucial cell fate decisions.

### Distinct megakaryocytic and erythroid progenitors can be isolated on the basis of *Runx1 P2-hCD4* expression

**Fig 5:**
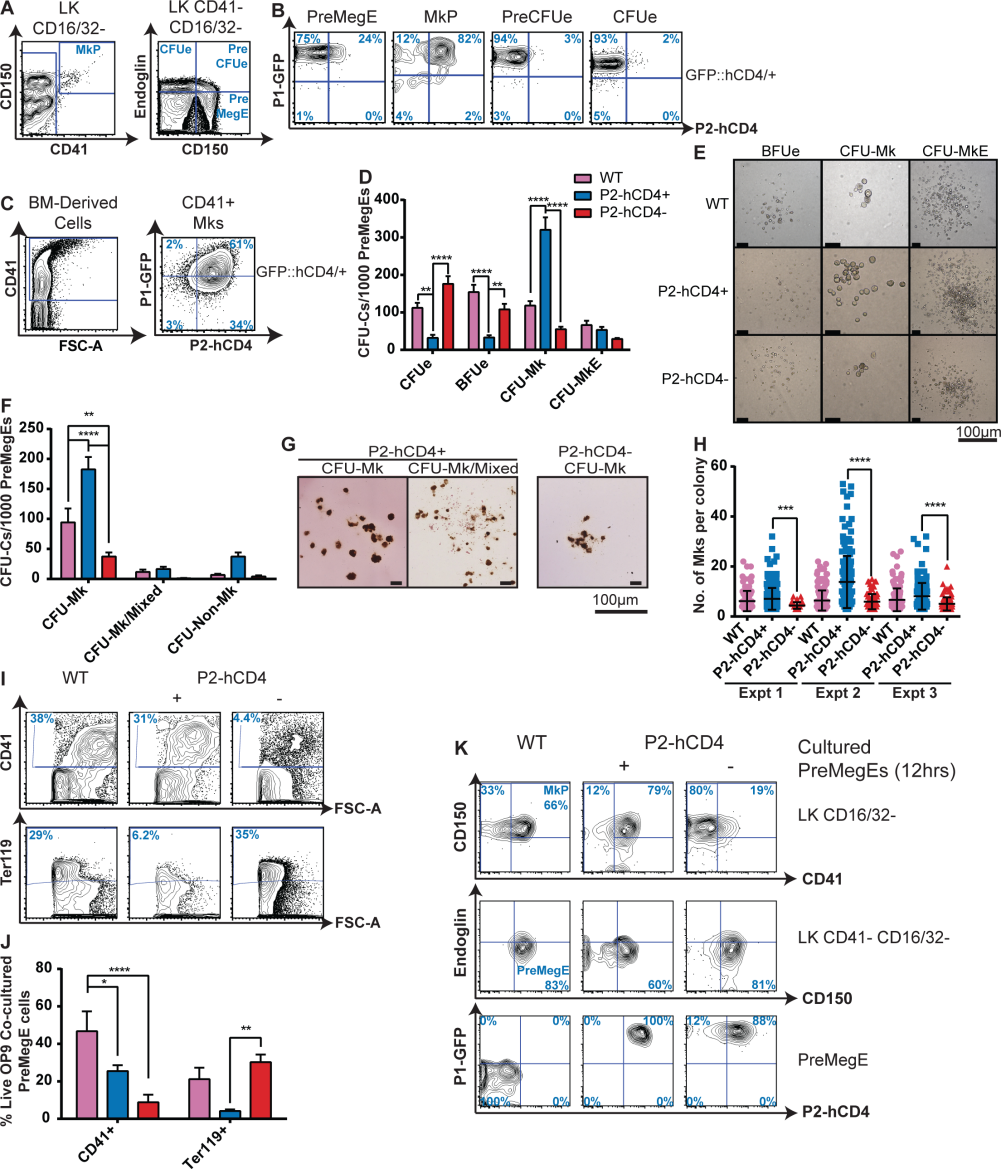
Distinct megakaryocytic and erythroid progenitors can be isolated on the basis of *Runx1 P2-hCD4* expression. (A) Contour plots of adult BM LK Mk/Ery progenitors. The PreMegE, MkP, PreCFUe and CFUe populations can by distinguished on the basis of cKit, CD41, SLAMF1 (CD150), CD16/32 and Endoglin cell surface expression. (B) Representative FACS plots of P1-GFP/P2-hCD4 expression in PreMegE, MkP, PreCFUe and CFUe subsets. (n = 8). (C) P1-GFP/P2-hCD4 expression in BM MkP-derived cultured CD41^+^ megakaryocytes. (n = 4). (D–H) CFU-C activity of *WT*, *P1*^*+*^ *P2*^*-*^ and *P1*^*+*^ *P2*^*+*^ PreMegEs. Cells were cultured either in pro-myeloid semi-solid methylcellulose-based medium (D) or in pro-megakaryocytic collagen-based MegaCultTM medium (F). Photographs of representative PreMegE-derived methylcellulose (E) and MegaCultTM (G) colonies. (n = 5) (H) Numbers of megakaryocytes per MegaCultTM CFU-Mk colony from 3 independent experiments (mean ± SD, Mann-Whitney *U* test). (I) Contour plots of OP9 co-cultured PreMegE cells isolated on day 7 and stained with CD41 and Ter119 antibodies. (J) Quantification of CD41^+^ megakaryocytes and Ter119^+^ erythrocytes in the PreMegE/OP9 co-culture assays. (n = 5). (K) Representative FACS plots of PreMegE cells following short-term (12 hours) culture in pro-myeloid liquid medium. Top: CD150/CD41 expression of LK CD16/32^-^ progenitor cells. Middle: Endoglin/CD150 expression of LK CD41 negative CD16/32 negative progenitors. Bottom: *P1-GFP*/*P2-hCD4* expression of immunophenotypic PreMegE (LK CD41- CD16/32- Endoglin- CD150^+^) cells (n = 3).

Erythropoiesis and megakaryopoiesis are highly similar developmental pathways, sharing numerous regulatory factors particularly at the point of lineage specification [33,34]. However, there are key differences and the specificity of a megakaryocyte maturation defect in *Runx1*-null adult BM implicates RUNX1 as a central player in Mk/Ery lineage determination [14]. Moreover, our observation that *P2-hCD4*^*-*^ and *P2-hCD4*^*+*^ MPPs have distinct Mk/Ery potential led us to investigate *Runx1* promoter activity in Mk/Ery-restricted progenitors further (Fig 5A). We observed that erythroid restricted PreCFUe and CFUe progenitors expressed solely *P1-GFP* whereas the MkP was chiefly *P1-GFP*^*+*^ *P2-hCD4*^*+*^ (Fig 5B). Because mature megakaryocytes are scarce in adult mice, BM-derived megakaryocytes were obtained by culturing purified MkPs *in vitro*. CD41-expressing megakaryocytes expressed *P2-hCD4* and a large fraction (60%) co-expressed *P1-GFP* (Fig 5C). Whilst lineage-restricted megakaryocytic and erythroid progenitors were highly homogeneous, the PreMegE fraction, which generates the MkP and PreCFUe populations, was markedly more heterogeneous; approximately 75% express solely *P1-GFP* whereas the remaining 25% were *P1-GFP*^*+*^ *P2-hCD4*^*+*^ (Fig 5B).

When compared to the relative homogeneity of the monopotent MkP, PreCFUe and CFUe populations, the heterogeneity of the PreMegE led us to consider the possibility of two functionally distinct and prospectively isolatable PreMegE subsets. We subsequently observed that erythroid CFU-C activity (CFUes and erythroid blast-forming units (BFUes)) was significantly enriched in the *P2-hCD4*^*-*^ PreMegE fraction compared to the *P2-hCD4*^*+*^ population (Fig 5D and 5E). By comparison, megakaryocyte CFU-C potential was highly enriched in the *P2-hCD4*^*+*^ fraction (Fig 5D–5H). In the MkP population, the *P2-hCD4*^*+*^ fraction possessed similar megakaryocytic CFU-C activity to its WT counterpart (S6A Fig), suggesting *Runx1* haploinsufficiency did not significantly impair megakaryocyte colony formation. In addition to being more numerous, CFU-Mks derived from *P2-hCD4*^*+*^ PreMegEs were also larger than those derived from the *P2-hCD4*^*-*^ fraction, the median number of cells per colony being doubled (Fig 5E, 5G and 5H). To determine whether this was a result of increased proliferation in the *P2-hCD4*^*+*^ PreMegE fraction, we analyzed their cell cycle status by measuring 5’ethynyl-2’-deoxyuridine (EdU) incorporation and DNA content (S6B Fig). More *P2-hCD4*^*+*^ PreMegE cells were in the EdU^+^ DNA Synthesis (S) phase compared to their *P2-hCD4*^*-*^ counterparts, suggesting *P2*-driven RUNX1B expression may confer a proliferative advantage on PreMegE cells.

The distinct megakaryocytic and erythroid potential of the two PreMegE fractions was further confirmed following co-culture with OP9 cells. After 7 days, *P2-hCD4*^*+*^ PreMegE cultures contained significantly more CD41^+^ megakaryocytes and significantly fewer Ter119^+^ erythroid cells than the *P2-hCD4*^*-*^ PreMegE cultures (Fig 5I and 5J). In addition, we performed clonal analyses by plating single *P2-hCD4*^*-*^ and *P2-hCD4*^*+*^ PreMegEs with OP9 (S6C Fig). This demonstrated that although the two fractions had similar clonal output (28% and 24% positive wells respectively) the *P2-hCD4*^*+*^ fraction contained more bi-potent megakaryocytic/erythroid progenitors (42.9% versus 25% “Mk + Ery”) and more monopotent megakaryocyte-producing progenitors (31.4% versus 12.5% “Mk only”) than the *P2-hCD4*^*-*^ fraction. The *P2-hCD4*^*-*^ fraction was highly enriched for monopotent erythroid-producing progenitors (62.5% “Ery only” compared to 25.7% in the *P2-hCD4*^*+*^ co-cultures). Therefore, as in the immature LSK HSPC compartment, upregulation of *P2* expression in PreMegEs appeared to coincide with a loss of erythroid and an enrichment of megakaryocytic specification.

To decipher their relative positions in the hematopoietic hierarchy, *P2-hCD4*^*-*^ and *P2-hCD4*^*+*^ PreMegE cells were cultured for up to 12 hours and analyzed (Fig 5K). We observed that *P2-hCD4*^*+*^ PreMegEs made a more rapid transition to an MkP immunophenotype than *P2-hCD4*^*-*^ cells. In addition *P2-hCD4*^*-*^ PreMegEs gave rise to both *P2-hCD4*^*-*^ and *P2-hCD4*^*+*^ fractions *in vitro*, whereas the *P2-hCD4*^*+*^ fraction did not appear to downregulate *P2-hCD4*. Taken together, these data suggest the *P2-hCD4*^*-*^ PreMegE can be placed earlier in the hematopoietic hierarchy, giving rise to the *P2-hCD4*^*+*^ PreMegE. Interestingly, we also observed that cultured *P2-hCD4*^*+*^ LSK48F^-^ cells produced only *P2-hCD4*^*+*^ immunophenotypic PreMegEs (S4B Fig). The differences in megakaryocytic and erythroid lineage potential in the *P2-hCD4* negative and positive LSK48F^-^ fractions may therefore be due to the preferential downstream specification of distinct PreMegE subpopulations.

### Global gene expression analysis of *Runx1 P2-hCD4* positive and negative PreMegEs enables the identification of WT equivalents

**Fig 6:**
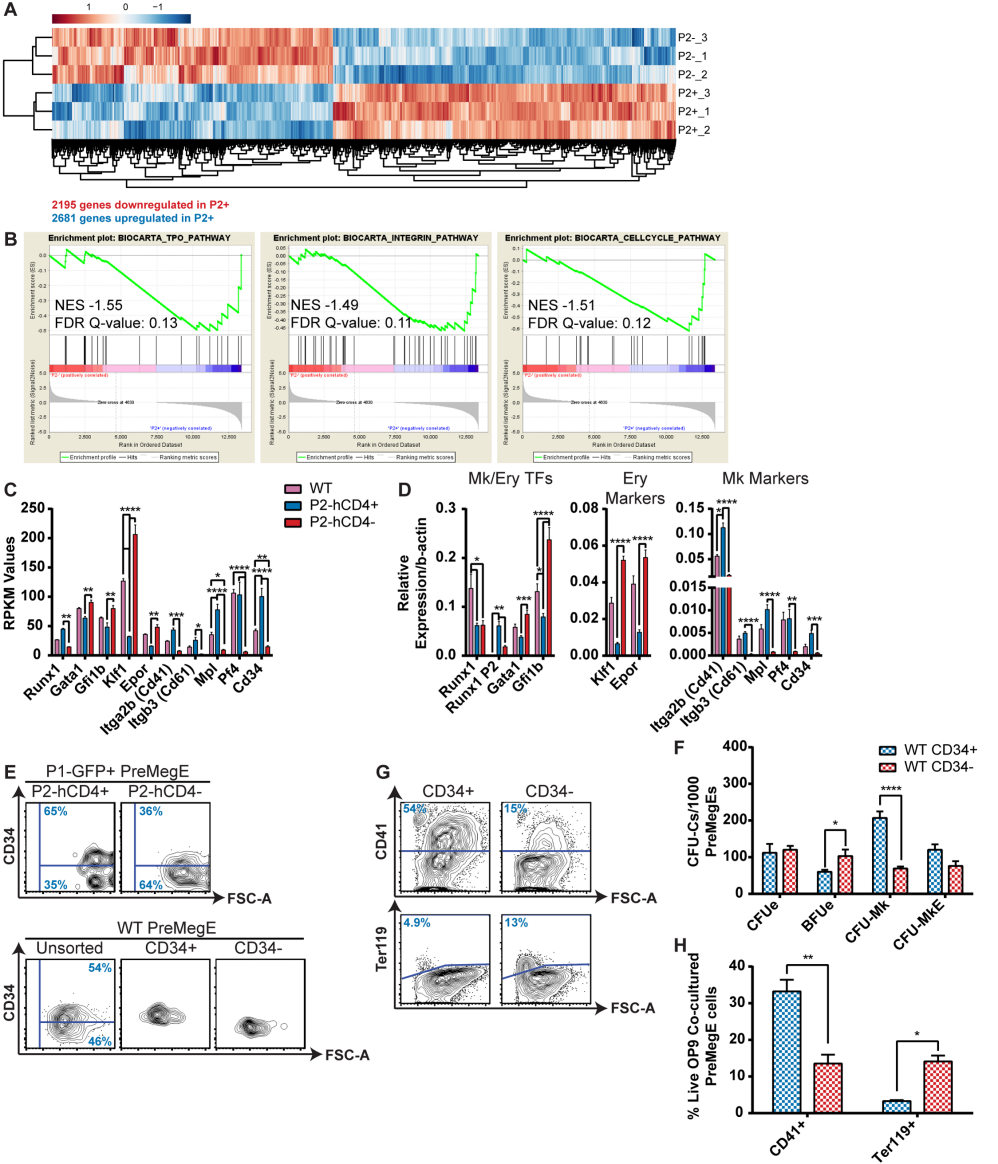
Global gene expression analysis of *Runx1 P2-hCD4+* and *P2-hCD4-* PreMegEs enables identification of WT equivalents. (A) Heat map depiction of genes at least 2-fold differentially expressed between P1^+^ P2^-^ (P2^-^) and P1^+^ P2^+^ (P2^+^) PreMegE samples, as determined by RNA Seq. Genes in red are upregulated and genes in blue are downregulated. (B) GSEA showing significantly enriched signaling pathways in the gene set upregulated in P2^+^ PreMegEs relative to P2^-^ PreMegEs. (C) Reads per kilobase per million mapped reads (RPKM) values of selected Mk/Ery- associated genes. (n = 3). (D) Quantitative PCR (qPCR) validation of expression of genes depicted in D. (n = 5). (E) Representative FACS plots of CD34 expression in P2^+^ and P2^-^ PreMegEs (top) and unsorted, purified CD34^+^ and purified CD34- WT PreMegEs (bottom). (*P1-GFP*::*P2-hCD4* mice n = 3; *WT* mice n = 5). (F) CFU-C activity of CD34^+^ and CD34^-^ PreMegEs following culture in pro-myeloid semi-solid methylcellulose-based medium. (n = 5). (G) Contour plots of OP9 co-cultured CD34^+^ and CD34^-^ PreMegE cells isolated on day 7 and stained with CD41 and Ter119 antibodies. (H) Quantification of CD41^+^ megakaryocytes and Ter119^+^ erythrocytes in the PreMegE/OP9 co-culture assays (n = 4).

To explore the distinct gene regulatory mechanisms involved in the bifurcation of the Mk/Ery pathway, and to identify candidate genes which may serve as markers to isolate the progenitors in WT BM, we performed global gene expression analysis by RNA-Seq (Fig 6A and S4 Table). The expression patterns of WT, *P2-hCD4*^*+*^ (*P2+*) and *P2-hCD4*^*-*^ (*P2-*) PreMegE samples were clearly separated based on principal component analysis, with WT cells clustering between the *P2+* and *P2-* samples (S7A Fig). When directly comparing the *P2+* and *P2-* populations, 4876 genes were found to be at least 2-fold differentially expressed (false discovery rate <0.05), 2681 being upregulated in *P2+* and 2195 in *P2-* PreMegEs (Fig 6A and S4 Table). Gene Set Enrichment Analyses (GSEA) revealed a significant correlation between *P2-hCD4* expression and activation of the Thrombopoietin (TPO) and Integrin pathways, both of which are crucial for megakaryopoiesis (Figs 6B and S7B–S7D) [35–37]. In line with the observed increased proliferative capacity of *P2-hCD4*^*+*^ PreMegEs, cell cycle regulators were also enriched in this population. Ingenuity pathway analysis (IPA) identified cell migration and blood cell recruitment as highly enriched functions and integrin signaling as the most significant activated pathway in *P2-hCD4*^*+*^ PreMegEs (S7E and S7G Fig). By contrast, functions and pathways associated with cell death and cell cycle inhibition were highly enriched in *P2-hCD4*^*-*^ PreMegEs (S7F and S7H Fig).

To further validate the distinct “pro-megakaryocytic” and “pro-erythroid” phenotypes of each PreMegE population, we screened, and validated by qPCR, the RNA Seq data for the expression of known Mk/Ery regulators and markers (Fig 6C and 6D). Early erythroid-associated factors, including Kruppel-like factor 1 (*Klf1*) and the Erythropoietin receptor (*Epor*) were significantly upregulated in *P2-hCD4*^*-*^ PreMegEs whereas numerous megakaryocyte-specific markers (Integrin alpha 2b (*Itga2b* or *Cd41*), Integrin beta 3 (*Itgb3* or *Cd61*), Myeloproliferative Leukemia Virus Oncogene (*Mpl*) and Platelet Factor 4 (*Pf4*)) were enriched in the *P2-hCD4*^*+*^ fraction. Interestingly, the transcription factors GATA binding protein 1 (*Gata1*) and Growth Factor Independent 1B (*Gfi1b*) were upregulated in *P2-hCD4*^*-*^ PreMegEs. Both factors are crucial for the normal development of both megakaryocytic and erythroid lineages: deletion of either *Gata1* or *Gfi1b* results in an early block in erythropoiesis at the PreCFUe stage whereas megakaryocytic maturation is impaired resulting in the accumulation of undifferentiated megakaryoblasts [38–41]. It would therefore appear that high *Gfi1b* and/or *Gata1* expression promote erythroid specification whereas lower levels would favor megakaryocytic commitment, but ultimately an increase of both would be required for megakaryocytic maturation and thrombopoiesis. It is therefore highly likely that differential expression of *Gata1* and *Gfi1b* at the PreMegE stage plays a role in Mk/Ery lineage determination and their differential expression may be driven by *P2*-driven RUNX1B.

In order to distinguish “pro-megakaryocytic” and “pro-erythroid” PreMegE subsets in WT mice by alternative means to our reporter line, we screened the list of differentially expressed genes in *P2-hCD4*^*+*^ and *P2-hCD4*^*-*^ PreMegEs for cell surface markers with commercially available antibodies validated for use in flow cytometry (S8A Fig). The majority of selected markers had low RPKM values, with the exception of *Itgb3* (*Cd61*) and *Cd34* (Figs 6C–6E and S8B). However, CD61 protein expression was not detected on *P2-hCD4*^*+*^ and *P2-hCD4*^*-*^ PreMegE cells by flow cytometry (S8C and S8D Fig). By contrast, CD34 expression was approximately 2-fold higher in *P2-hCD4*^*+*^ PreMegEs compared to *P2-hCD4*^*-*^ cells, both in terms of numbers of positive cells and median fluorescence intensity (MFI; Figs 6E and S8D). WT CD34^+^ and CD34^-^ PreMegEs were therefore FACS sorted to >95% purity (Fig 6E) and their lineage output and *Runx1* isoform expression elucidated. Importantly, *Runx1 P2* expression was substantially higher in the CD34^+^ PreMegEs compared to the CD34^-^ fraction (S3 Fig). Interestingly, *P1* activity was also increased in the CD34^+^ cells, resulting in a 20% increase in total *Runx1* expression. It is therefore unclear how important expression of the RUNX1B isoform is for the promotion of megakaryopoiesis compared to enhanced RUNX1 expression overall. Indeed, *P2* transcripts were even more highly expressed in WT MkPs, contributing to the highest levels of total *Runx1* in all analyzed HSPCs (S3C Fig). However, *P1* expression was in fact decreased in MkPs compared to PreMegEs, offering additional evidence in favor of a specific pro-megakaryopoiesis role for the *P2-*specified RUNX1B protein.

Akin to *P2-hCD4*^*+*^ PreMegEs, WT CD34^+^ PreMegEs had enhanced CFU-Mk and diminished BFUe activity compared to CD34^-^ cells (Fig 6F). Myeloid co-culture with OP9 stromal cells confirmed these phenotypes, as CD34^+^ PreMegEs produced substantially more CD41^+^ megakaryocytes and fewer Ter119^+^ erythroid cells (Fig 6G and 6H). Single-cell OP9 co-culture revealed the CD34^+^ PreMegE compartment was highly enriched for monopotent “Mk only” progenitors (79.2% versus 41.7%) and bipotent “Mk + Ery” progenitors (20.8% versus 8.3%) compared to the CD34^-^ fraction (S8E Fig). By contrast, monopotent “Ery-only” progenitors accounted for 50% of the CD34^-^ PreMegE cultures but were apparently absent from the CD34^+^ fraction. We have therefore established the existence of prospectively isolatable “pro-megakaryocytic” CD34^+^ and “pro-erythroid” CD34^-^ PreMegE cells in WT mice.

### Deletion of the dominant RUNX1C isoform does not ablate adult hematopoiesis but results in numerous lineage-specific defects

**Fig 7:**
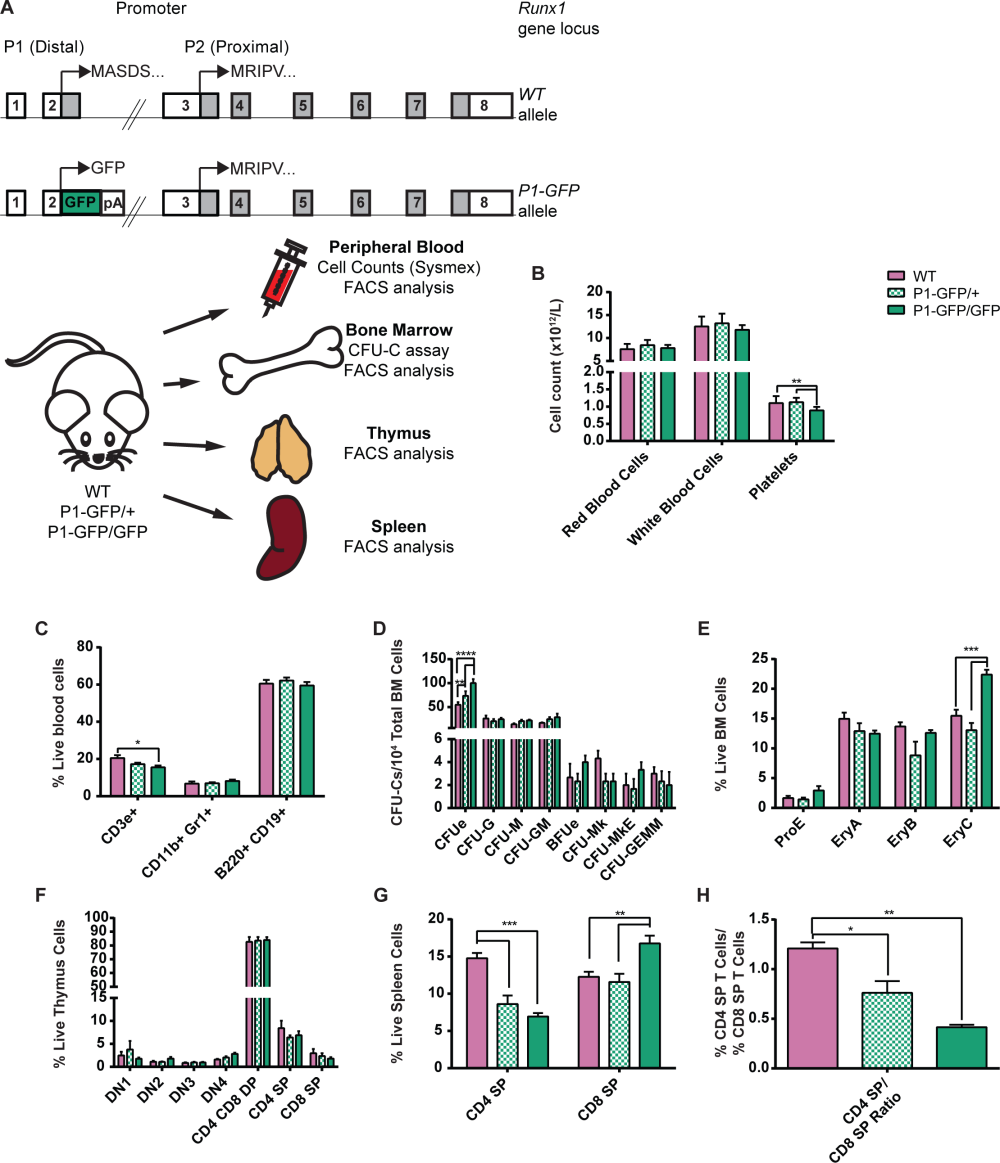
Impact of the absence of *P1*-directed *Runx1* expression on adult hematopoiesis. (A) Top: Schematic diagrams of the *Runx1 WT* (top) and *P1-GFP* (bottom) alleles. Expression of GFP is directed by *Runx1* promoter *P1* but expression of *Runx1* from the *P2* promoter remains intact. Bottom: Schematic diagram of the experimental design for the investigation of the impact of *Runx1 P1* deletion on adult hematopoiesis. Peripheral blood, BM, thymus and spleen samples were collected from adult WT, *P1-GFP* heterozygous (*P1-GFP/+*) and homozygous (*P1-GFP/GFP*) adult mice. All samples were analyzed for mature blood cell surface marker expression. In addition, blood samples were subjected to automated cell counts (Sysmex) and CFU-C assays were performed on unfractionated BM. (B) Peripheral blood cell counts of WT, *P1-GFP/+* and *P1-GFP/GFP* mice as determined by Sysmex automated cell counting. (C) Numbers of CD3e+ T cells, CD11b+ Gr1+ GM cells and B220+ CD19+ B cells as a proportion of total ACK-lysed blood cells from WT, *P1-GFP/+* and *P1-GFP/GFP* mice. (D) CFU-C activity of WT, *P1-GFP/+* and *P1-GFP/GFP* unfractionated ACK-lysed BM following culture in pro-myeloid semi-solid methylcellulose-based medium. (n = 4.) (E) Numbers of erythroid lineage (ProE, EryA, EryB and EryC) cells as a proportion of live unfractionated BM cells. (F) Numbers of T cell lineage populations as a proportion of live unfractionated thymus cells. (G) Numbers of CD4 SP and CD8 SP T cells as a proportion of live unfractionated spleen cells. (H) Ratio of splenic CD4 SP T cells to splenic CD8 SP T cells (n = 4).

Having established that the *P1-*directed RUNX1C isoform is expressed throughout adult hematopoiesis, we decided to determine how its absence would impact the overall homeostasis of the adult blood system. Previously, we utilized the *P1-GFP* homozygous mouse to investigate the requirement for RUNX1C at the onset of hematopoiesis and found it to be dispensable for hematopoietic commitment [29]. However, this may be due to *P2* being the dominant promoter at this stage. We therefore analyzed hematopoietic populations in adult WT, RUNX1C heterozygous (*P1-GFP/+*) and homozygous knockout (*P1-GFP/GFP*) mice (Fig 7A). Despite the high expression of *P1* in erythroid, myeloid and lymphoid progenitors, we observed no significant perturbation of circulating red or white blood cell numbers upon performing automated cell counts (Fig 7B). Erythroid differentiation appeared to be normal, as peripheral blood hematocrit, hemoglobin concentration and the reticulocyte counts of the RUNX1C null mice were unaltered compared to their WT and heterozygous littermates (S9A–S9C Fig). Similarly, myelo-lymphoid cell fate decisions did not appear to be significantly affected, as the proportions of circulating monocytes, neutrophils and lymphocytes were unaffected (S9D-F). However, a modest but significant decrease in platelet numbers was observed in RUNX1C null mice compared to both the WT and heterozygous animals (Fig 7B). Their plateletcrit was also slightly decreased (albeit not to a significant extent) but the mean platelet volume was unaltered (S9G and S9H Fig). This suggests that, unlike in the conditional total *Runx1* null adult mouse model, platelet maturation is not impaired but specification may be hampered.

FACS analysis of circulating blood cells and BM confirmed the presence of equal proportions of CD11b^+^ Gr1^+^ GM lineage and B220^+^ CD19^+^ B lymphoid cells in WT, *P1-GFP/+* and *P1-GFP/GFP* mice (Figs 7C and S10). However, the numbers of CD3ε^+^ T cells were significantly reduced, suggesting that the absence of RUNX1C partially impairs T cell specification. We therefore analyzed the thymic T cell populations in greater detail and found that CD4/8 DN, DP and SP population numbers were not altered in *P1-GFP/GFP* mice (Fig 7F). However, the ratio of CD4 SP:CD8 SP T cells in the spleen was severely perturbed, as *P1-GFP/GFP* mice had considerably fewer CD4 SP and more CD8 SP T cells compared to WT littermates (Fig 7G and 7H). This therefore suggests that RUNX1C is dispensable for the DN to DP transition, observed to be blocked in total *Runx1* deficient mice [13]. Nonetheless, the RUNX1C knockout recapitulates the defect in CD4 SP and CD8 SP T cell specification observed in total *Runx1+/-* mice, clearly demonstrating an important role for *P1*-driven RUNX1 activity in the T cell lineage [4,42,43].

To determine whether the absence of *P1-*directed RUNX1C expression impacts adult colony-forming HSPC populations, we performed myeloid CFU-C assays on unfractionated BM from WT, *P1-GFP/+* and *P1-GFP/GFP* mice (Fig 7D). GM, MkE and multilineage GEMM colony numbers were unaffected, but RUNX1C null BM cells produced significantly more erythroid CFUe colonies than either the WT or *P1-GFP/+* cultures. FACS analysis of unlysed BM revealed a significant expansion of the EryC population in the RUNX1C null mice, a stage which coincides with almost complete silencing of both the *Runx1 P1* and *P2* promoters (Fig 7E). In combination with the observed mild thrombocytopenia, it appears that the absence of RUNX1C may favor erythroid specification over megakaryopoiesis, a phenotype observed recently in mouse and human HSPCs depleted for total RUNX1 [44]. Overall, *P1-*directed RUNX1C activity may be dispensable for normal adult hematopoiesis but its absence nonetheless results in defects reminiscent of total RUNX1 deficiency.

### Expression of the *AML1-ETO9a* oncogene preferentially induces *Runx1 P2* expression

**Fig 8:**
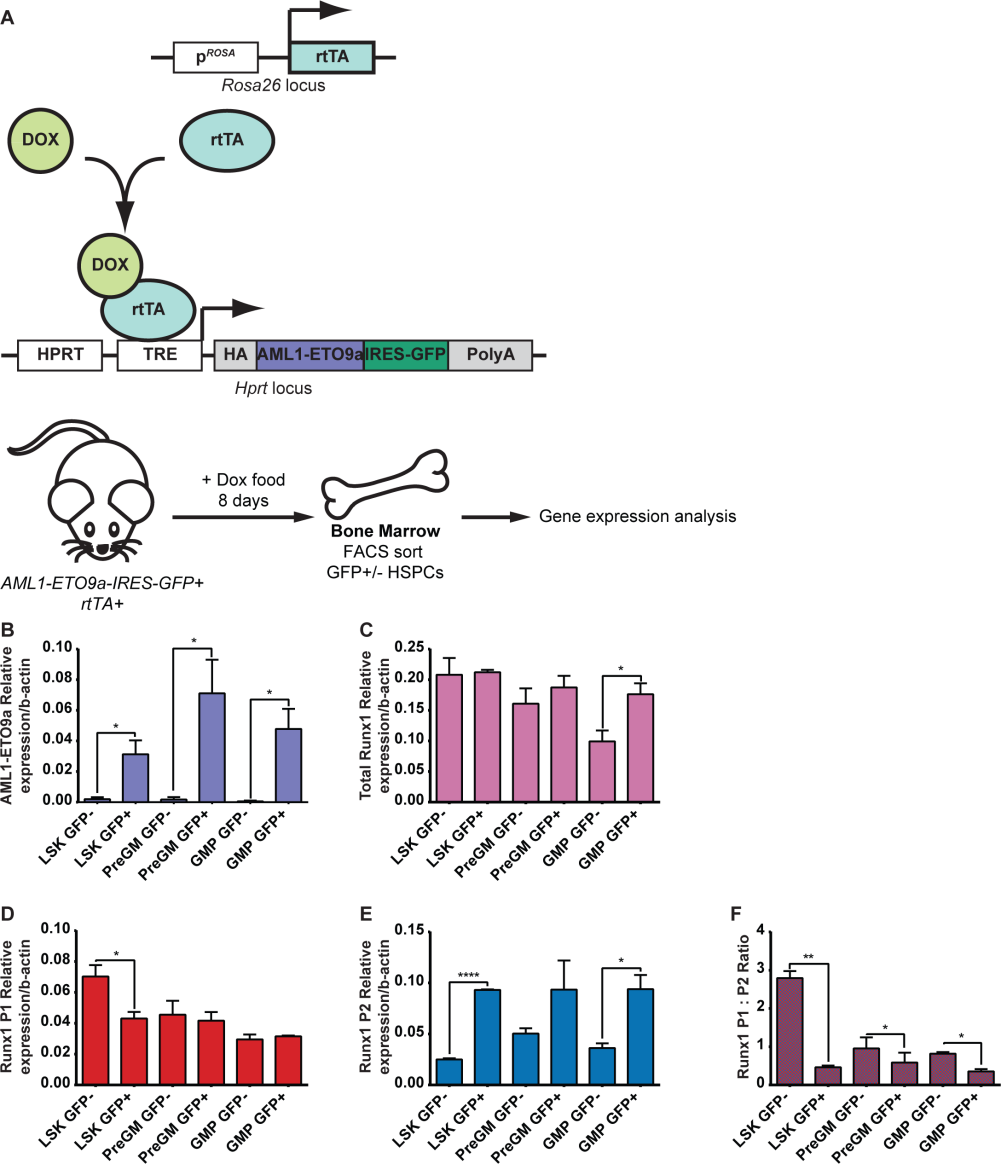
Effect of short-term induction of *AML1-ETO9a* expression on *Runx1* expression in BM HSPCs. (A) Top: Schematic representation of the *Rosa26* and *Hprt* loci in the Doxycycline-inducible AML1-ETO9a GFP mouse model. The reverse tetracycline-controlled transcriptional activator (rtTA) is constitutively expressed under the control of the *Rosa* promoter. Upon binding to Doxycycline, the rtTA is capable of binding to and activating a tetracycline responsive element (TRE) located in the ubiquitously expressed *Hprt* locus, resulting in expression of the hemagglutinin (HA)-tagged AML1-ETO9a::IRES-GFP construct also incorporated into this locus. Bottom: Schematic diagram of the experimental design to induce short-term expression of *AML1-ETO9a* in adult mice by administering Doxycycline in food for 8 days. Bone marrow cells were the harvested and GFP^+^ and GFP^-^ HSPC populations were isolated by FACS sorting for RNA extraction and gene expression analysis. (B-E) Gene expression analysis by qPCR of *AML1-ETO9a* (B), total *Runx1* (C), *Runx1 P1* (D) and *Runx1 P2* (E). (F) Ratio of *Runx1 P1*:*P2* expression (n = 3).

Increasingly it is becoming apparent that, in addition to a more classically defined tumor suppressor role, WT RUNX1 is required for the promotion of leukemogenesis in certain leukemia subtypes. Notably, AML1-ETO-driven CBF AML appears to be dependent on maintaining WT RUNX1 activity [21,22]. However, although AML1-ETO appears to promote *RUNX1* expression, it is unclear whether AML1-ETO oncogene expression promotes the expression of one *Runx1* promoter over another [45]. To address this question, we utilized a novel mouse model expressing a Doxycycline-inducible *AML1-ETO9a* transgene under the control of a Tetracycline Responsive Element (TRE, Fig 8A). The *AML1-ETO9a* oncogenic transcript is expressed in a majority of t(8;21) AML patients studied and encodes a truncated AML1-ETO protein with enhanced leukemogenic potential [46,47]. We therefore took advantage of our ability to induce *AML1-ETO* expression in adult mice (by administering Doxycycline in the food for 8 days) and studied the impact on *Runx1* isoform expression *in vivo* by isolating AML1-ETO-expressing (AML1-ETO9a-IRES-GFP^+^) and non-expressing (AML1-ETO9a-IRES-GFP^-^) BM HSPCs and quantitating *Runx1* expression through qRT-PCR (Fig 8A). This allowed us to study the effect of *AML1-ETO* expression on WT *Runx1* expression as one of the earliest events at the initiation of leukemogenesis.

Firstly, we confirmed the presence and absence of *AML1-ETO9a* expression in BM GFP^+^ and GFP^-^ HSPCs respectively (Fig 8B). We chose to analyze LSK, PreGM and GMP cells as the immature HSPC and GM-lineage progenitors contain the leukemia propagating cell fraction in numerous AML patient samples and in a previously described *AML1-ETO* mouse model [48,49]. Whilst in the PreGM, and GMP *Runx1 P1* expression was unperturbed by the expression of *AML1-ETO*, it was in fact decreased by approximately 40% in LSK GFP^+^ cells compared to GFP^-^ (Fig 8D). In all three HSPC populations, however, the presence of *AML1-ETO* resulted in an upregulation of *Runx1 P2* expression (Fig 8E), albeit not to a significant extent in PreGM cells. This resulted in an increase in total *Runx1* expression in the GMP fraction (Fig 8C) but also a significant decrease in the *P1*:*P2* ratio in all three populations, particularly in the LSK compartment, a phenotype associated with enhanced CFU-C activity, particularly in the GM lineage (Fig 8F).

## Discussion

**Fig 9:**
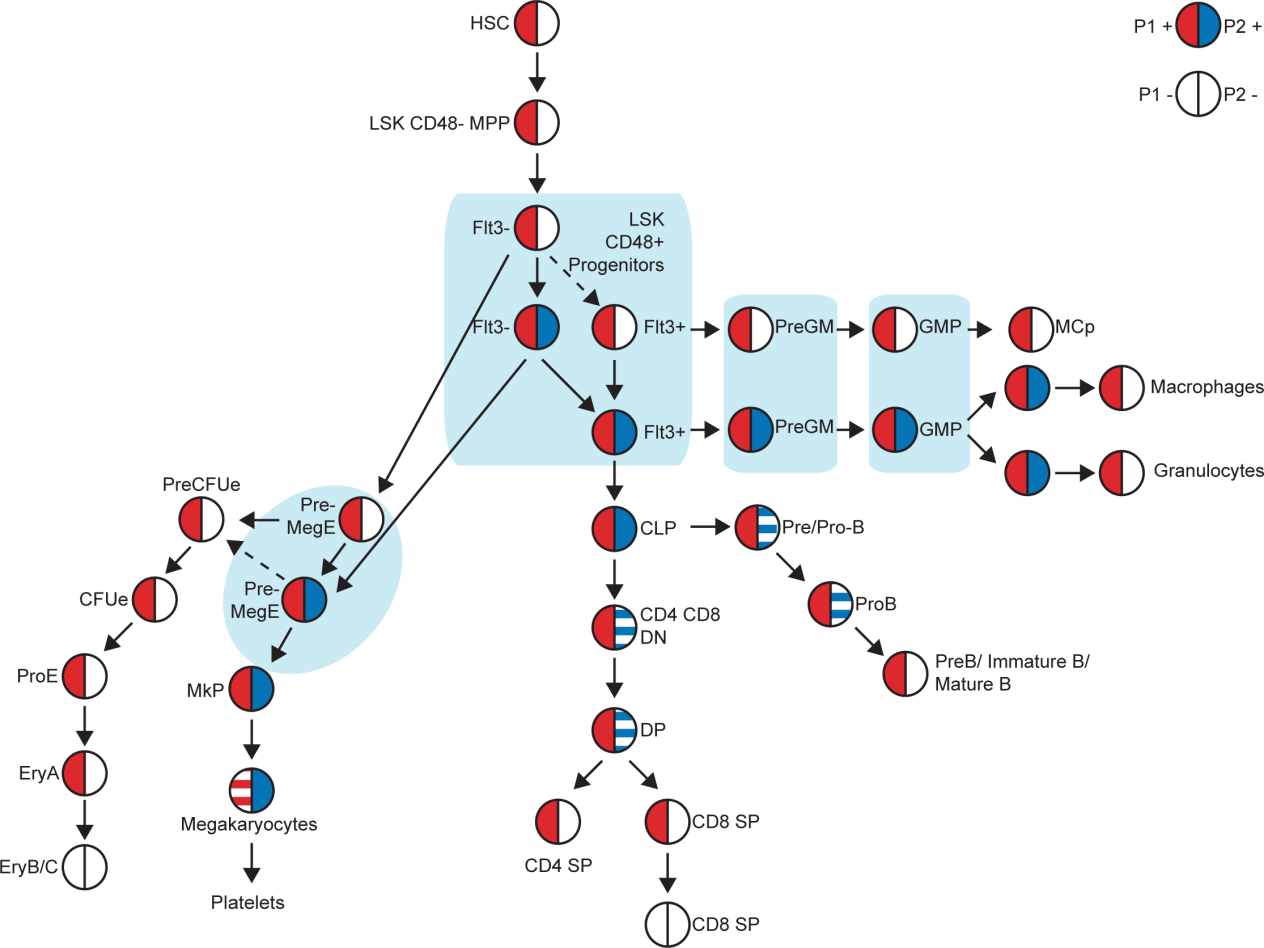
Model of *Runx1 P1* and *P2* expression in adult megakaryocytic/erythroid lineages. *Runx1 P1* (red) and *P2* (blue) expression in adult hematopoietic stem, progenitor and lineage positive cells, as determined using the *P1-GFP*::*P2-hCD4* double knock-in mouse model.

Our understanding of the hematopoietic hierarchy, and of the complexity of cell fate decisions in this system, has been increasingly refined in recent years. For a long time, it was assumed that the most mature shared ancestor for all myeloid populations was the Common Myeloid Progenitor (CMP), until this population was subsequently dissected and shown to be a heterogeneous population containing the PreGM and PreMegE fractions [50,51]. Using the *Runx1 P1-GFP*::*P2-hCD4* dual reporter mouse model, we have now similarly demonstrated further heterogeneity in the PreMegE fraction, prospectively isolating “pro-erythroid” *P2*^*-*^ and “pro-megakaryocytic” *P2*^*+*^ fractions (Fig 9). Moreover, we have successfully identified their equivalents in WT BM as being CD34^-^ and CD34^+^ respectively. CD34, a cell-cell adhesion factor previously characterized as a direct RUNX1 transcriptional target [52] and expressed on vascular-associated tissue and selected HSPCs, was previously used to distinguish the CMP from the Megakaryocyte/Erythroid Progenitor (MEP) [50]. By *in vitro* cell tracing experiments, we have determined that the *P2*^*+*^ PreMegE lies directly downstream of the *P2*^*-*^ PreMegE, apparently contradicting a CMP-based model as this involves downregulation of CD34 expression prior to Mk/Ery lineage commitment. Moreover, we have demonstrated immunophenotypic *P2*^*-*^ PreMegEs can be directly derived from *P2*^*-*^ LSK48F^-^ MPPs, lending weight to the argument that progenitors lose Mk/Ery potential before separation of the GM and lymphoid pathways [32]. In fact, our model goes further, proposing that erythroid potential is downregulated prior even to megakaryocytic potential, either coinciding with or as a direct result of *Runx1 P2* upregulation.

Interestingly, comparative analysis of transcription factor binding motifs by rVISTA [53–56] in the vicinity of the *P1* and *P2* regions revealed the presence of conserved erythroid transcription factor EKLF (KLF1) motifs in the *P1* region but none surrounding *P2* (S11A and S11B Fig). By contrast, FLI1-binding motifs are present in both regions. This is interesting as RUNX1 has recently been implicated in regulating the balance of EKLF and FLI1 activity, which promote Ery and Mk output respectively [44]. In addition, EKLF and FLI1 may in fact act upstream of RUNX1, for example with EKLF directly activating *Runx1 P1* but not *P2* expression, a state which is reinforced by the enhanced EKLF expression in *P1+P2-* pro-erythroid PreMegEs compared to *P1*^*+*^*P2*^*+*^ pro-megakaryocytic PreMegEs. Analysis of ChIP-Seq data from the mouse ENCODE project [53,54,57,58] also reveal some interesting differences in GATA1 and SCL (TAL1) binding to the *P1* and *P2* promoter regions in megakaryocytes and erythroblasts (S11C Fig). GATA1 and TAL1 binding appear largely unchanged in the vicinity of the *P1* promoter in both cell types. By contrast, GATA1 binding is observed at *P2* and GATA1+TAL1 binding approximately 15kb upstream in erythroblasts but not megakaryocytes. It is conceivable, therefore, that GATA1-mediated transcriptional repression of the *P2* promoter occurs in the erythroid lineage, whereas the absence of a GATA1-containing complex enables its derepression and recruitment of activating factors instead.

The high number of differentially expressed genes (>4000) in the *P2*^*-*^ and *P2*^*+*^ PreMegEs lends credence to the hypothesis that they are derived from distinct progenitor ancestors. Commitment to megakaryocytic or erythroid lineages may even occur earlier, at the HSC level; the *P2*^*-*^ and *P2*^*+*^ MPPs may themselves be derived from pro-erythroid and pro-megakaryocyte HSCs respectively as previously described [59–61]. Regardless of this, the increased purification of phenotypically distinct progenitors within the hematopoietic hierarchy afforded by our model will enable the investigation of molecular mechanisms involved in cell fate decisions with significantly greater precision. In fact the role of RUNX1 in lineage commitment was recently expanded to include promotion of megakaryopoiesis over erythropoiesis through repression of *KLF1* [44]. Overexpression studies were performed solely using a RUNX1B construct and knockdown was non-isoform specific, so it remained unclear how important the isoform specificity is to the process of megakaryocytic or erythroid lineage commitment. Our investigation of the *P1-GFP/GFP* model suggests RUNX1C plays a specific role in these lineages, as its absence means circulating platelet numbers are decreased whereas BM CFUes and EryCs are increased. However, we cannot discount the fact that this phenotype may be due to an overall decrease in RUNX1 protein as opposed to the specific loss of RUNX1C and therefore further studies utilizing either targeted mutagenesis of the two *Runx1* promoters separately or isoform-specific knockdown whilst not impacting the overall level of RUNX1 would be required to explore this possibility.

As previously mentioned, *P1* is the dominant promoter in adult hematopoiesis, being active in all *Runx1*-expressing populations. *P2* expression is far more heterogeneous, confined to immature/progenitor subsets of the GM and lymphoid lineages and megakaryocytes. With the exception of megakaryocytes, it appears that downregulation of *P2* is a prerequisite of terminal differentiation of these lineages. We also observed that, at least in myeloid lineages, *P2*-expression correlates with enhanced CFU-C activity and in the PreMegE specifically with increased proliferation. Numerous cell cycle regulators are upregulated in *P2*^*+*^ PreMegEs, several of which have previously been identified as putative RUNX1-targets. A unique feature of megakaryocytic differentiation is polyploidisation achieved through undergoing numerous abortive cell cycles. Cell cycle activators are therefore highly expressed in these cells, as is *Runx1 P2*. It is also of interest that *P2* expression has previously been observed in newly emerging embryonic HSC-containing hematopoietic clusters but not in the more quiescent BM HSC populations [28]. Despite their distinct roles in hematopoiesis, many parallels have been drawn between the specification of HSCs and megakaryopoiesis [62]. HSCs and MkPs share similar cell surface marker profiles and have numerous regulatory pathways in common [63,64]. These include critical dependence on TPO signaling and hematopoietic transcription factors, including the CBF complex, Ets and HOX-related genes, several of which are upregulated in *P2+* PreMegEs (S4 Table) [62,65]. Megakaryocytes appear to have more in common with embryonic than adult HSCs, their production being characterized by CD41 expression and RUNX1-dependency [7,66]. Our observations therefore suggest that expression of *P2-*driven RUNX1B may actively promote cell cycling, with a role in expanding HSPC numbers and is then downregulated to allow terminal differentiation of the B/T/GM and erythroid lineages. It would be of interest to investigate to what extent RUNX1B, and also RUNX1C, indeed directly regulate different transcriptional targets and the mechanisms through which they may achieve this.

In addition to erythroid progenitors, mast cell progenitor specification did not appear to require *P2* expression. Intriguingly, both are lineages which do not appear to be adversely affected by the absence of *Runx1*; complete ablation of *Runx1* in adult mice has no impact on peripheral red blood cell numbers, whereas mast cell development is normal in *Runx1 P1-*null mice [14,67]. Whether this suggests *Runx1* expression is entirely incidental in these lineages will need to be investigated further.

The requirement for WT RUNX1 activity in AML has been extensively studied in recent years. In AML1-ETO CBF AML in particular, a balance of AML1-ETO and RUNX1 expression must be maintained to promote stem cell gene expression and repress differentiation-associated gene expression [68]. Moreover, it appears AML1-ETO may directly regulate *Runx1* expression, as depletion of AML1-ETO leads to a decrease in *RUNX1* levels in Kasumi1 cells [45]. However, whether expression of *P1* or *P2* was favored in this context had not been investigated. By utilizing an inducible AML1-ETO mouse model, we were able to establish that AML1-ETO expression resulted in a specific upregulation of *Runx1 P2*. We have found *P2* expression coincides with enhanced CFU activity and proliferation in HSPCs. Ben-Ami *et al*. previously demonstrated RUNX1 enhances the viability of preleukemic AML1-ETO-expressing cells [21], therefore it may be that RUNX1B activity specifically enhances a preleukemic phenotype in emerging CBF AML leukemia propagating cells. Interestingly, Trombly *et al*. observed the recruitment of AML1-ETO to *P1* and the *+23* enhancer but not to *P2* in Kasumi1 cells [45]. Therefore, the mechanism of AML1-ETO’s activation of *P2* is of considerable interest. AML1-ETO may directly activate *P2*, potentially via the *+23* enhancer or it may instead promote expression of other transcriptional activators which enhance *P2* activity. Alternatively, AML1-ETO may directly repress *P1*, resulting in a compensatory upregulation of *P2* by a secondary mechanism. These possibilities will all need to be explored further.

## Materials and Methods

### Mice

*P1-GFP*::*P2-hCD4* and *P1-GFP* mice have previously been described [29]. The *AML1-ETO9a-IRES-GFP/rtTA* mice were generated as follows: HA-tagged AML1-ETO9a cDNAs (provided by the Zhang laboratory [47]) were subcloned into a tet-ON vector in front of an IRES-GFP as described [69]. Ainv18 ES cells [69] (which constitutively express the rtTA under the control of the *Rosa26* promoter) were then transfected with this tetracycline-inducible *AML1-ETO9a* construct by electroporation and stably transfected clones were selected with G418 (0.5mg/ml, Life Technologies) for 10–14 days. Chimeric mice were then generated by injecting *AML1-ETO9a-IRES-GFP/rtTA* ES cells into C57BL6J blastocysts. To induce *AML1-ETO9a-IRES-GFP* expression, 12 week-old mice were fed irradiated diet supplemented with 545mg/kg Doxycycline (ssniff Spezialdiäten GmbH) for 8 days prior to humane culling and tissue collection. All animal work was performed under regulations governed by UK Home Office Legislation under the Animals (Scientific Procedures) Act 1986. Details of animal husbandry and tissue collection are listed in S1 File.

### Flow cytometry analysis and cell sorting

Dead cells were excluded using either 0.5μg/ml 7-Aminoactinomycin D (7-AAD, eBioscience) or 1μg/ml Hoechst 33258 (Life Technologies). Biotinylated antibody staining was detected by a secondary incubation step with fluorochrome-conjugated Streptavidin. Prior to flow sorting of HSPCs, bone marrow cells stained with biotinylated anti-lineage antibodies were lineage-depleted using anti-biotin-conjugated magnetic beads (Miltenyi) and then stained with additional antibodies, including conjugated streptavidin. Red blood cell depletion was performed by treatment with ACK lysis buffer (154mM ammonium chloride, 9.99mM potassium bicarbonate, 0.110mM EDTA) for 5 minutes at room temperature, followed by quenching with Phosphate-Buffered Saline (PBS).

Details of flow cytometry antibodies and reagents are listed in S1 Table. Details of flow cytometry antibody combinations used for each analysis or sort are listed in S2 Table.

For cell cycle analysis, *in vivo* EdU incorporation was performed by injecting 1.125mg EdU dissolved in PBS intraperitoneally into adult mice. After two hours, bone marrow was harvested and stained with hematopoietic stem and progenitor cell surface markers as detailed in S2 Table. Cells were then stained using the Click-iT EdU Alexa Fluor 647 Flow Cytometry Assay Kit (Life Technologies). Total DNA was stained with 1μg/ml FxCycle Violet Stain (Life Technologies).

Cells were analyzed using a LSR-II or LSR-II Fortessa analyzer, a FACSAria-II cell sorter or a FACSAria-III cell sorter (BD).

### Automated peripheral blood counts

Tail vein blood (no more than 50μl per mouse) was sampled from 12 week old mice using heparinized end-to-end Micro Pipettes (Vitrex) and analyzed on a Sysmex XT 2000i analyzer, according to the manufacturer’s instructions.

### Cell culture

#### OP9 and OP9-DL1 co-culture

Cells were cultured in 5% CO_2_ and 5% O_2_ at 37°C. Mouse OP9 stromal cells were maintained in Alpha-MEM (Lonza) supplemented with 10% fetal bovine serum (FBS, Sigma-Aldrich), 2mM L-glutamine (Gibco), 1x Penicillin-Streptomycin (Sigma-Aldrich) and were routinely tested for mycoplasma contamination. For myeloid OP9 co-culture PreMegE and LSK48F- MPPs were seeded on mouse OP9 stromal cells (approximately 5000/ml) at a density of approximately 2000 cells/ml in IMDM (Lonza) supplemented with 10% Fetal Bovine Serum (FBS) for Mouse Myeloid Colony-Forming Cells (StemCell Technologies), 0.45mM monothioglycerol (MTG, Sigma-Aldrich), 2mM L-glutamine (Gibco), 1x Penicillin-Streptomycin (100U/ml Penicillin, 100μg/ml Streptomycin, Sigma-Aldrich), 2U/ml erythropoietin (Eprex, Janssen-Cilag Ltd), and medium conditioned by cell lines producing IL3, TPO and Stem Cell Factor (SCF) (1% final concentration). PreMegEs were cultured for 7 days and LSK48F- MPPs for 8 days before being harvested and stained for myeloid cell surface markers (TER119, CD41, GR1 and CD11B as detailed in S2 Table). For single cell co-culture, single PreMegE cells were directly sorted into 96-well plate wells containing 100μl of the above medium and approximately 500 OP9 cells and cultured for 7 days before positive wells were assessed by microscopy and FACS analyzed as above.

For B cell and T cell co-culture LSK48F- MPPs were seeded on mouse OP9 and OP9-DL1 stromal cells respectively at a density of approximately 2000 cells/ml in IMDM supplemented with 20% FBS (Harlan), 2mM L-glutamine, 1x Penicillin-Streptomycin, 5ng/ml FLT3L (PeproTech) and 1ng/ml IL7 (PeproTech). Cultures were passed into fresh media every 4–5 days for 21 days (with IL7 concentrations reduced to 0.25ng/ml in OP9-DL1 co-cultures from day 12 onwards) and harvested and stained for B and T cell surface markers (CD45R (B220), CD19, CD4 and CD8A as detailed in S2 Table).

#### Short-term culture of HSPCs

Purified PreGM, GMP and PreMegE cells were cultured at 37°C in 5% CO_2_ and atmospheric O_2_ for 6–18 hours in pro-myeloid medium (IMDM supplemented with 10% FBS for Mouse Myeloid Colony-Forming Cells, 10% Protein-free hybridoma medium (PFHM-II, Gibco), 180μg/ml Transferrin (Roche Diagnostics), 0.45mM MTG, 50ng/ml ascorbic acid (Sigma-Aldrich), 2mM L-glutamine, 1x Penicillin-Streptomycin, 4U/ml erythropoietin, 5ng/ml IL11 (R&D Systems), 10ng/ml IL6 (R&D Systems), 10ng/ml M-CSF (R&D Systems) and medium conditioned by cell lines producing IL3, GMCSF, TPO and SCF (1% final concentration)). Purified LSK48F- MPPs were cultured for 12–24 hours in Alpha MEM (Lonza) supplemented with 10% FBS, 0.45mM MTG, 2mM L-glutamine, 1x Penicillin-Streptomycin, 1U/ml erythropoietin, and medium conditioned by cell lines producing IL3, TPO and SCF (1% final concentration)) (adapted from [70]). Cells were then harvested and stained with either LSK HSPC markers or myeloid progenitor markers as described in S2 Table.

For longer-term culture (7–11 days), PreGM and GMP cells were cultured in pro-myeloid medium as described above before being harvested and stained with GM and mast lineage markers (CD11B, GR1, F4/80, C-KIT and FCΕR1Α as detailed in S2 Table).

Sorted MkP cells were cultured for 4 days in pro-myeloid medium (see above).

#### Hematopoietic colony-forming assays

Methylcellulose colony-forming assays were performed as previously described [71], except 10% (FBS) (StemCell Technologies) was added in place of the Fetal Bovine Plasma-derived Serum Platelet Poor (PDS). 200 PreMegEs or 50 LSK48F- MPPs were plated per dish in duplicate. CFUes were scored after 3–4 days and other colonies after 8 days under a microscope (DM IL; Leica). Megakaryocyte-specific colony-forming assays were performed by plating 1000 MkPs or PreMegEs in MegaCult^TM^ medium (StemCell Technologies). Cells were cultured, fixed, stained and analyzed according to the manufacturer’s protocols.

### Gene expression

#### Quantitative PCR

RNA was extracted using the RNeasy Plus Micro Kit (QIAGEN). Complementary DNA was synthesized using the SuperScript III First-Strand Synthesis System (Life Technologies). Quantitative PCR (qPCR) was performed using Universal ProbeLibrary assays (Roche); primers and probes are listed in S3 Table. With the exception of TER119+ erythroid and CD11b+ GR1+ GM lineage cells, expression values were normalized to *beta-actin* (*b-actin*). Owing to the significant variation of *b-actin* in these lineages, total *Runx1* expression in these compartments was calculated relative to input and normalized to the level in CD11b+ GR1+ cells. To determine the relative contributions of *Runx1 P1* and *P2* transcripts to total *Runx1* expression, the relative efficiencies of the qPCR primers were calculated in a titration experiment using known quantities of *P1* and *P2*-expressing plasmid template DNA.

#### RNA sequencing

Tissues from 3 mice were pooled per sample. Total RNA was extracted from purified PreMegEs as described above. Indexed PolyA libraries were prepared using 50ng of total RNA and 16 cycles of amplification in the Agilent SureSelect Strand-Specific RNA Library Prep Kit for Illumina Sequencing (Agilent). Libraries were quantified by qPCR using a KAPA Library Quantification Kit for Illumina platforms (Kapa Biosystems Inc.). Paired-end 75bp sequencing was carried out by clustering 1.7pM of the pooled libraries on a NextSeq 500 sequencer (Illumina Inc.) for 3 biological replicates per population. Details of sequence data analysis are given in S1 File.

The data discussed in this publication have been deposited in NCBI’s Gene Expression Omnibus [72] and are accessible through GEO series accession number GSE68958.

### Statistical analysis

Flow cytometry plots display the mean values of each indicated population. Unless otherwise indicated, data were evaluated using an Ordinary 2-way ANOVA and expressed as mean ± standard error of the mean (SEM). *P*<0.05 was considered statistically significant.

*P<0.05, **P<0.01, ***P<0.001, ****P<0.0001

## Supporting Information

S1 Fig Immunophenotypic characterization of BM, spleen and thymus hematopoietic lineage positive populations. (Related to Fig 2) (A–F) Contour plots of lineage marker expression in adult BM (A, B, C), spleen (D and F) and thymus (E). (A) CD71/Ter119 expression of live cells (left) and CD71 expression/FSC of Ter119 high cells (right) in BM. (B) CD11b/Gr1 (left) and CD11b/F4/80 (right) expression in live BM cells. (C) B220/CD11b+CD3ε+Ter119 expression in live cells (left), cKit/CD19 expression in B220^+^ cells (middle) and IgM/IgD expression in B220^+^ CD19^+^ cKit^-^ cells (right) in BM. (D) B220/CD19 expression in live cells (left) and IgM/IgD expression in B220^+^ CD19^+^ cells (right) in spleen. (E) CD4/CD8a expression in live cells (left) and CD25/CD44 expression in CD4 CD8 double negative (DN) cells (right) in thymus. (F) CD4/CD8a expression in live spleen cells.
(PDF)

S2 Fig Comparative *Runx1* expression in WT mature lineage positive populations. (Related to Fig 2) (A-C) Gene expression analysis of *Runx1 P1* and *Runx1 P2* as a proportion of total *Runx1* in erythroid and GM (A), B (B) and T (C) cell lineage populations isolated from WT BM, spleen and thymus. (n = 3.) Owing to the high variability in *b-actin* expression in mature erythroid and GM blood cells, gene expression values for these populations were normalized to input and expressed relative to CD11b+ Gr1+ cells. Otherwise gene expression is depicted relative to *b-actin*.
(PDF)

S3 Fig *Runx1* expression in WT BM HSPCs. (Related to Figs 3–6) (A-C) Gene expression analysis of total *Runx1* (A), *Runx1 P1* (B) and *Runx1 P2* (C) in Lin- cKit+ HSPC populations isolated from WT BM, normalized to *b-actin* (n = 3).
(PDF)

S4 Fig *In vitro* tracing of *WT* and *P1-GFP*::*P2-hCD4/+* LSK48F- hematopoietic progenitors. (Related to Fig 3) Representative FACS plots of LSK48F^-^ MPPs following 18 hours *in vitro* myeloid culture. (A) Lin, cKit/Sca1, CD150/CD41, CD16/32 and Endoglin/CD150 expression of WT, P2^+^ and P2^-^ cultured LSK48F^-^ cells. (B) P1-GFP/P2-hCD4 expression of LSK48F^-^ derived immunophenotypic LSK and PreMegE cells (n = 3).
(PDF)

S5 Fig *In vitro* differentiation of *WT* and *P1-GFP*::*P2-hCD4/+* PreGM and GMP progenitors. (Related to Fig 4) (A–B) Top: FACS plots of CD11b/F4/80 and cKit/FcεR1α expression of day 11 cultured PreGM (A) and GMP (B) cells. Bottom: quantification of GM subsets following 7 and 11 days culture. (n = 4). (C) P1-GFP/P2-hCD4 expression in the BM Lin^-^ cKit^+^ Sca1^-^ FcεR1α^-^ CD27^-^ Ly6c^-^ Integrin Beta7^+^ MCp. (Representative of 3 independent experiments.) (D-E) Representative FACS plots of PreGMs (D) and GMPs (E) following 12 hours *in vitro* myeloid culture. (D) CD16/32/CD150, Endoglin/CD150 and P1-GFP/P2-hCD4 expression of PreGM-derived cells. (E) CD16/32/Cd150 and P1-GFP/P2-hCD4 of GMP-derived cells. (n = 3)
(PDF)

S6 Fig Cell cycle and clonal analysis of *WT* and *P1-GFP*::*P2-hCD4/+* Mk/Ery Progenitors. (Related to Fig 5) (A) CFU-C activity of *WT* and *P1*^*+*^ *P2*^*+*^ MkPs following culture in MegaCultTM medium. (n = 5). (B) Cell cycle status of *WT*, *P1*^*+*^ *P2*^*-*^ and *P1*^*+*^ *P2*^*+*^ PreMegEs, as determined by in vivo EdU incorporation and DNA content analysis. (n = 3). (C) Table showing the clonal analysis of *P1*^*+*^ *P2*^*-*^ and *P1*^*+*^ *P2*^*+*^ PreMegEs. Shown are the numbers of positive wells at day 7 of OP9 co-culture relative to the numbers of single PreMegE cells plated on day 0 and the proportion of wells that contained CD41^+^ cells (Mk only), Ter119^+^ cells (Ery only) or CD41^+^ and Ter119^+^ cells (Mk + Ery). Data are compiled from 5 independent experiments.
(PDF)

S7 Fig Global gene expression analysis of *P2-* and *P2+* PreMegEs. (Related to Fig 6) (A) Principal component analysis of the RNA Seq expression data from WT, P2^-^ and P2^+^ PreMegE cells. (B-D) Heat maps depicting expression of gene sets from GSEA plots (displayed in Fig 6B) in P2^-^ and P2^+^ PreMegE cells. (E–F) Diseases/functions upregulated (E) or downregulated (F) in P2^+^ PreMegEs compared to P2^-^ PreMegEs as determined by IPA. (G–H) Signaling pathways activated (G) or inhibited (H) in P2^+^ PreMegEs compared to P2^-^ PreMegEs as determined by IPA.
(PDF)

S8 Fig Candidate *P2+* PreMegE cell surface markers and clonal analysis of CD34+/- WT PreMegEs. (Related to Fig 6) (A) Log fold change (counts) of cell surface markers in P2^+^ PreMegEs compared to P2^-^ PreMegEs as determined by RNA Seq. (B) RPKM values of cell surface markers in WT, P2^-^ and P2^+^ PreMegEs. (n = 3). (C) Representative FACS plot of CD61 expression in P2^+^ and P2^-^ PreMegEs. (n = 2). (D) Ratio of median fluorescence intensity (MFI) and percentage of positive cells for CD61 and CD34 protein expression in P2^+^ PreMegEs compares to P2^-^ PreMegEs, determined by FACS. (n = 2). (E) Table showing the clonal analysis of CD34^+^ and CD34^-^ *WT* PreMegEs. Shown are numbers of positive wells at day 7 of OP9 co-culture relative to the numbers of single PreMegE cells plated on day 0 and the proportion of wells that contained CD41^+^ cells (Mk only), Ter119^+^ cells (Ery only) or CD41^+^ and Ter119^+^ cells (Mk + Ery). Data are compiled from 3 independent experiments.
(PDF)

S9 Fig Additional parameters derived from Sysmex analysis of peripheral blood from *Runx1 P1* null adult mice. (Related to Fig 7) (A-H) Quantitation of Hematocrit (A), Total hemoglobin concentration (B), Reticulocyte count (C), Monocyte count (D), Neutrophil count (E), Lymphocyte count (F), Plateletcrit (G) and Mean Platelet Volume (H) in peripheral blood of *WT*, *P1-GFP/+* and *P1-GFP/GFP* mice. (n = 4)
(PDF)

S10 Fig FACS analysis of mature GM and B cell lineage cells in *Runx1 P1* null adult mice. (Related to Fig 7) (A—B) Numbers of GM (A) and B (B) lineage cells as a proportion of live ACK-lysed BM cells. (C) Numbers of B lineage cells as a proportion of live ACK-lysed spleen cells (n = 4).
(PDF)

S11 Fig *In silico* analysis of potential Mk/Ery transcription factor binding motifs in the *Runx1 P1* and *P2* promoter regions. (A-B) rVISTA analysis of EKLF, FLI1, GATA1 and TAL1 binding motifs located in regions surrounding *Runx1 P1* (A) and *Runx1 P2* (B). Top, Blue: All motifs found in the mouse loci. Top, Green: Motifs conserved between mouse and human. Middle: Genetic conservation between mouse and human loci. Bottom: Schematic of mouse *Runx1* locus, aligned to conservation map. (C) UCSC Browser tracks of mouse *Runx1* locus, featuring GATA1 and TAL1 ChIP-seq data (performed in megakaryocytes and erythroblasts) acquired by the Mouse ENCODE Project, aligned to UCSC *Runx1* transcripts and vertebrate genetic conservation data.
(PDF)

S1 Table Details of flow cytometry reagents. (DOCX)

S2 Table Antibody combinations used for FACS analysis and sorting. (DOCX)

S3 Table Primers and probes used for qPCR. (DOCX)

S4 Table Differentially expressed genes in *P2-* and *P2+* PreMegEs. (XLSX)

S1 File Supplemental methods and references. (DOCX)
