# Correction: RUNX1B Expression Is Highly Heterogeneous and Distinguishes Megakaryocytic and Erythroid Lineage Fate in Adult Mouse Hematopoiesis

**DOI:** 10.1371/journal.pgen.1006084

**Published:** 2016-05-20

**Authors:** Julia E. Draper, Patrycja Sroczynska, Olga Tsoulaki, Hui Sun Leong, Muhammad Z. H. Fadlullah, Crispin Miller, Valerie Kouskoff, Georges Lacaud

There is an error in the representation of white blood cell counts in Fig 7B. Specifically, the Y-axis label states the data is expressed as ³x10^12^/L² but the correct values should be expressed as ³x10^9^/L². The red blood cell counts and platelet count data in the rest of Fig 7B are correct. Please see the correct Fig 7 here.

**Fig 7:**
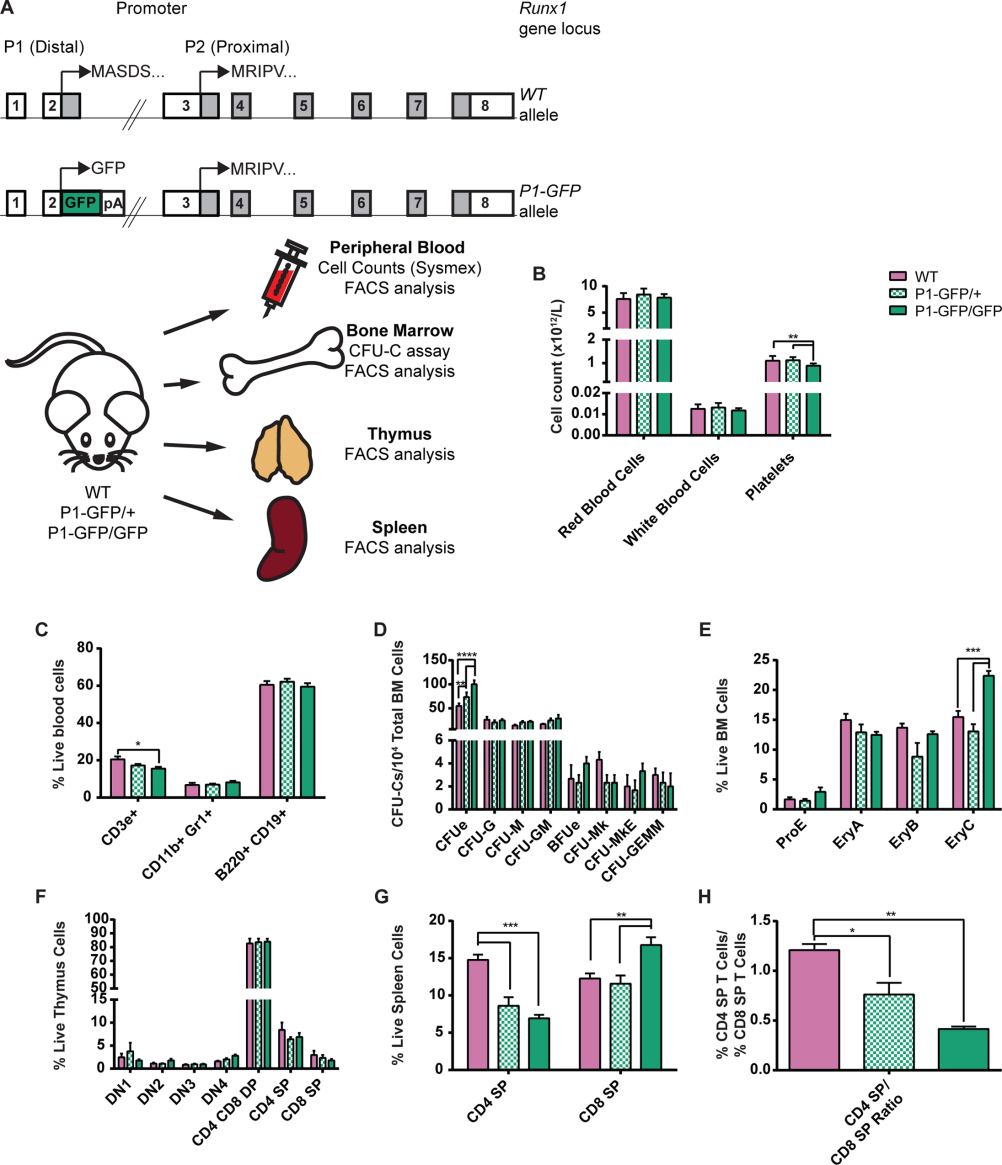
Impact of the absence of *P1*-directed *Runx1* expression on adult hematopoiesis. (A) Top: Schematic diagrams of the *Runx1 WT* (top) and *P1-GFP* (bottom) alleles. Expression of GFP is directed by *Runx1* promoter *P1* but expression of *Runx1* from the *P2* promoter remains intact. Bottom: Schematic diagram of the experimental design for the investigation of the impact of *Runx1 P1* deletion on adult hematopoiesis. Peripheral blood, BM, thymus and spleen samples were collected from adult WT, *P1-GFP*heterozygous (*P1-GFP/+*) and homozygous (*P1-GFP/GFP*) adult mice. All samples were analyzed for mature blood cell surface marker expression. In addition, blood samples were subjected to automated cell counts (Sysmex) and CFU-C assays were performed on unfractionated BM. (B) Peripheral blood cell counts of WT, *P1-GFP/+* and *P1-GFP/GFP* mice as determined by Sysmex automated cell counting. (C) Numbers of CD3e+ T cells, CD11b+ Gr1+ GM cells and B220+ CD19+ B cells as a proportion of total ACK-lysed blood cells from WT, *P1-GFP/+* and *P1-GFP/GFP* mice. (D) CFU-C activity of WT, *P1-GFP/+* and *P1-GFP/GFP*unfractionated ACK-lysed BM following culture in pro-myeloid semi-solid methylcellulose-based medium. (n = 4.) (E) Numbers of erythroid lineage (ProE, EryA, EryB and EryC) cells as a proportion of live unfractionated BM cells. (F) Numbers of T cell lineage populations as a proportion of live unfractionated thymus cells. (G) Numbers of CD4 SP and CD8 SP T cells as a proportion of live unfractionated spleen cells. (H) Ratio of splenic CD4 SP T cells to splenic CD8 SP T cells (n = 4).
