# Supplementary figures and images for: RUNX1B Expression Is Highly Heterogeneous and Distinguishes Megakaryocytic and Erythroid Lineage Fate in Adult Mouse Hematopoiesis
Source: PLoS Genet. 2016 Jan 25;12(1):e1005814. doi: 10.1371/journal.pgen.1005814 (PMC4726605; doi:10.1371/journal.pgen.1005814)

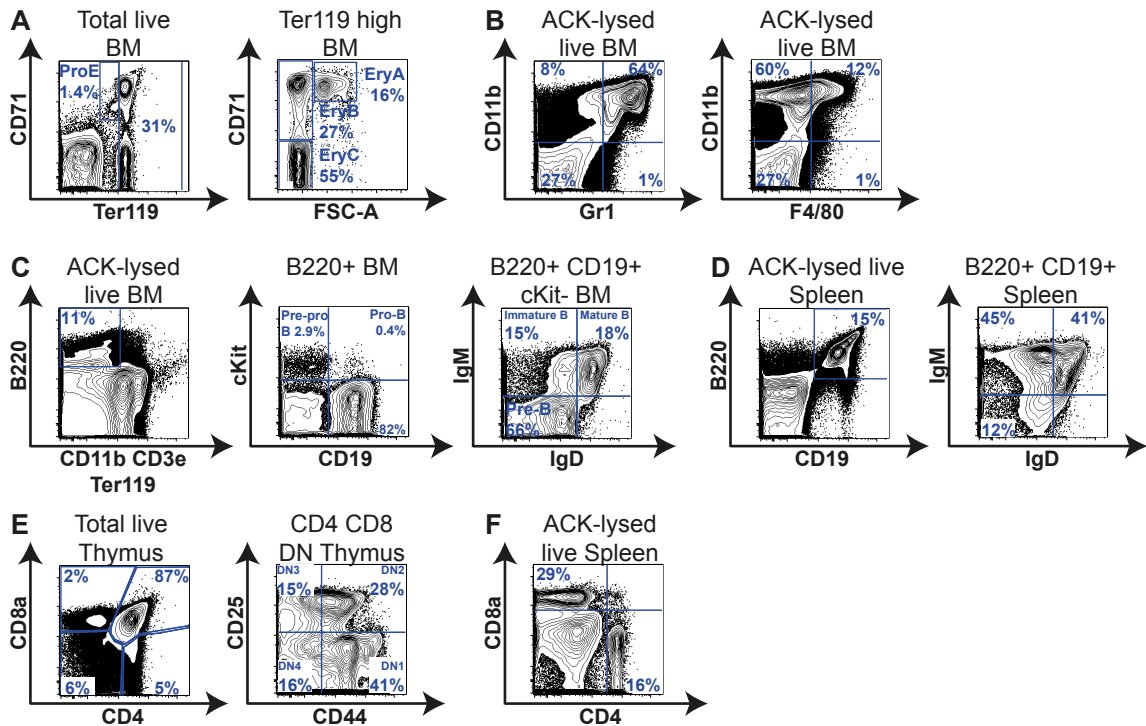

S1 Fig.

Supplement: S1 Fig — (Related to Fig 2) (A–F) Contour plots of lineage marker expression in adult BM (A, B, C), spleen (D and F) and thymus (E). (A) CD71/Ter119 expression of live cells (left) and CD71 expression/FSC of Ter119 high cells (right) in BM. (B) CD11b/Gr1 (left) and CD11b/F4/80 (right) expression in live BM cells. (C) B220/CD11b+CD3ε+Ter119 expression in live cells (left), cKit/CD19 expression in B220+ cells (middle) and IgM/IgD expression in B220+ CD19+ cKit- cells (right) in BM. (D) B220/CD19 expression in live cells (left) and IgM/IgD expression in B220+ CD19+ cells (right) in spleen. (E) CD4/CD8a expression in live cells (left) and CD25/CD44 expression in CD4 CD8 double negative (DN) cells (right) in thymus. (F) CD4/CD8a expression in live spleen cells. (PDF) [file pgen.1005814.s001.pdf]

**A**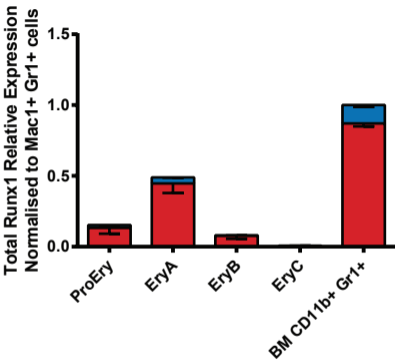**B**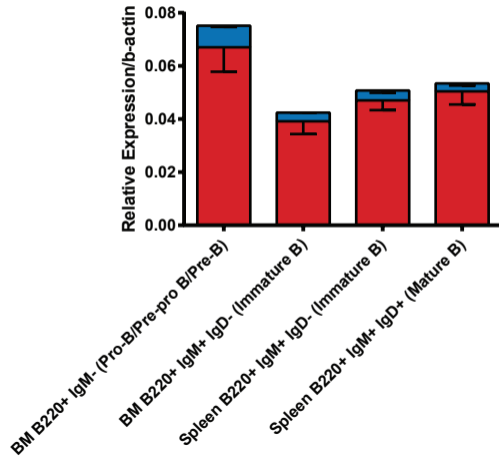**C**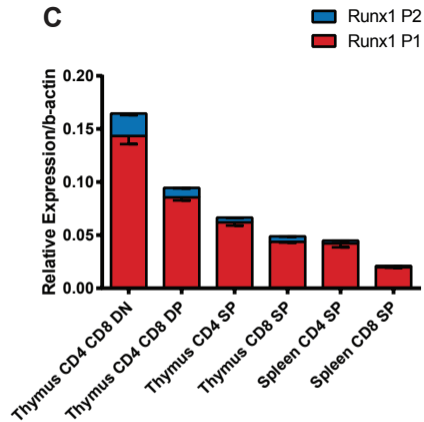**S2 Fig.**

Supplement: S2 Fig — (Related to Fig 2) (A-C) Gene expression analysis of Runx1 P1 and Runx1 P2 as a proportion of total Runx1 in erythroid and GM (A), B (B) and T (C) cell lineage populations isolated from WT BM, spleen and thymus. (n = 3.) Owing to the high variability in b-actin expression in mature erythroid and GM blood cells, gene expression values for these populations were normalized to input and expressed relative to CD11b+ Gr1+ cells. Otherwise gene expression is depicted relative to b-actin. (PDF) [file pgen.1005814.s002.pdf]

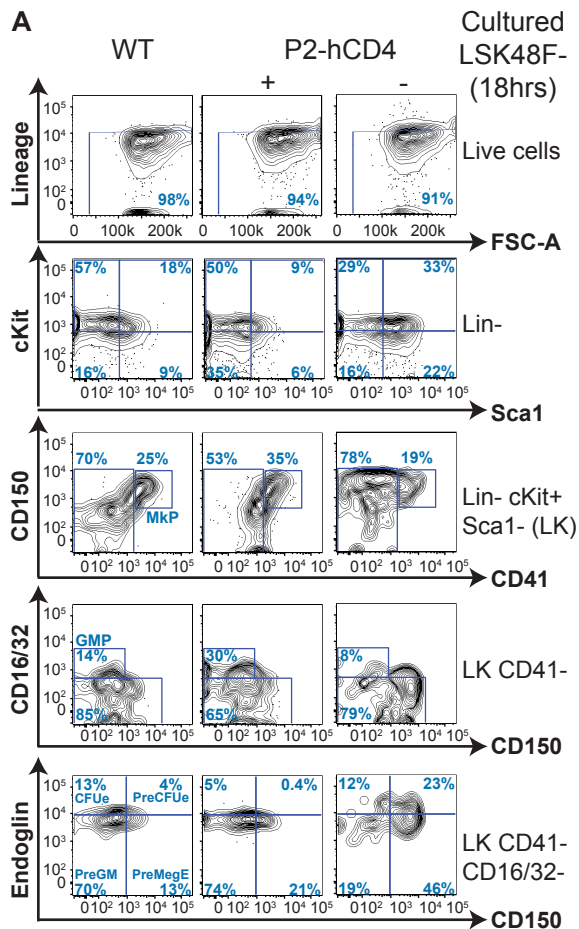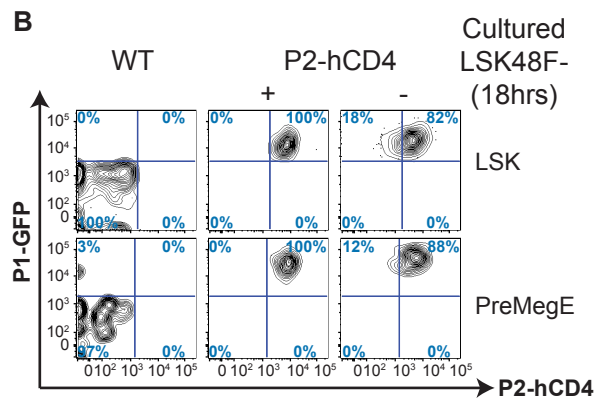

Supplement: S4 Fig — (Related to Fig 3) Representative FACS plots of LSK48F- MPPs following 18 hours in vitro myeloid culture. (A) Lin, cKit/Sca1, CD150/CD41, CD16/32 and Endoglin/CD150 expression of WT, P2+ and P2- cultured LSK48F- cells. (B) P1-GFP/P2-hCD4 expression of LSK48F- derived immunophenotypic LSK and PreMegE cells (n = 3). (PDF) [file pgen.1005814.s004.pdf]

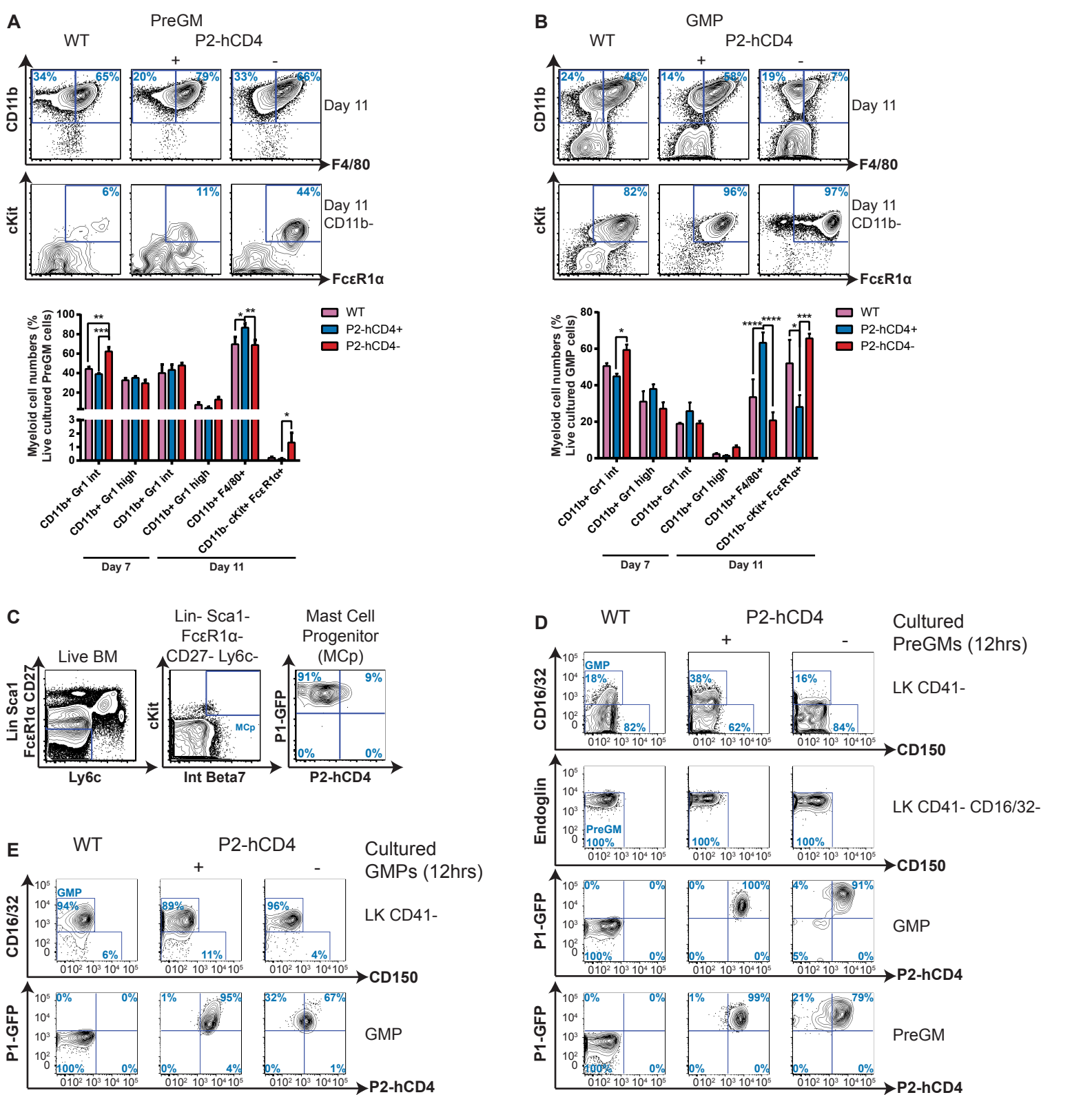

S5 Fig.

Supplement: S5 Fig — (Related to Fig 4) (A–B) Top: FACS plots of CD11b/F4/80 and cKit/FcεR1α expression of day 11 cultured PreGM (A) and GMP (B) cells. Bottom: quantification of GM subsets following 7 and 11 days culture. (n = 4). (C) P1-GFP/P2-hCD4 expression in the BM Lin- cKit+ Sca1- FcεR1α- CD27- Ly6c- Integrin Beta7+ MCp. (Representative of 3 independent experiments.) (D-E) Representative FACS plots of PreGMs (D) and GMPs (E) following 12 hours in vitro myeloid culture. (D) CD16/32/CD150, Endoglin/CD150 and P1-GFP/P2-hCD4 expression of PreGM-derived cells. (E) CD16/32/Cd150 and P1-GFP/P2-hCD4 of GMP-derived cells. (n = 3) (PDF) [file pgen.1005814.s005.pdf]

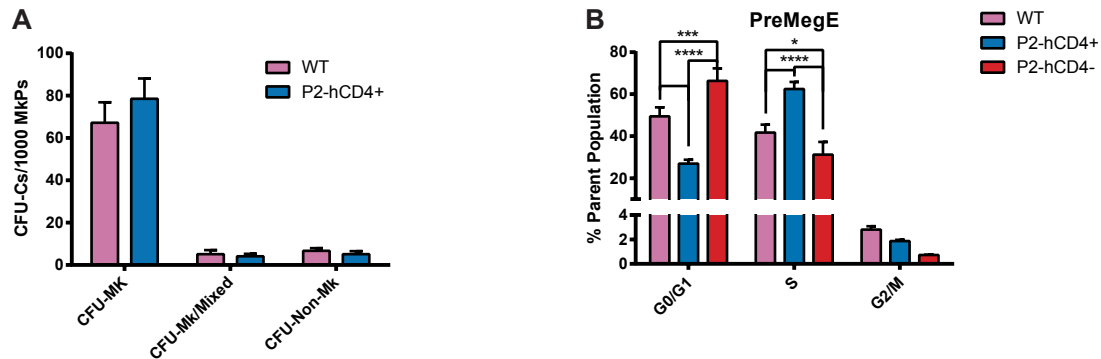

**C**

| <i>P1-GFP::P2-hCD4/+</i> PreMegE | No. cells per well | No. positive wells | % Mk only | % Ery only | % Mk + Ery |
|----------------------------------|--------------------|--------------------|-----------|------------|------------|
| <i>P1+ P2+</i>                   | 1                  | 117/480 (24%)      | 31.4      | 25.7       | 42.9       |
| <i>P1+ P2-</i>                   | 1                  | 133/480 (28%)      | 12.5      | 62.5       | 25         |

Supplement: S6 Fig — (Related to Fig 5) (A) CFU-C activity of WT and P1+ P2+ MkPs following culture in MegaCultTM medium. (n = 5). (B) Cell cycle status of WT, P1+ P2- and P1+ P2+ PreMegEs, as determined by in vivo EdU incorporation and DNA content analysis. (n = 3). (C) Table showing the clonal analysis of P1+ P2- and P1+ P2+ PreMegEs. Shown are the numbers of positive wells at day 7 of OP9 co-culture relative to the numbers of single PreMegE cells plated on day 0 and the proportion of wells that contained CD41+ cells (Mk only), Ter119+ cells (Ery only) or CD41+ and Ter119+ cells (Mk + Ery). Data are compiled from 5 independent experiments. (PDF) [file pgen.1005814.s006.pdf]

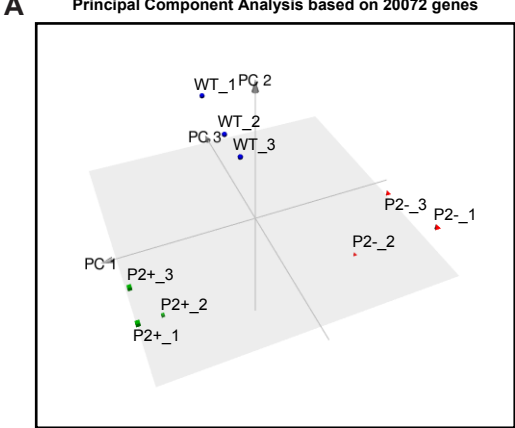

**B**

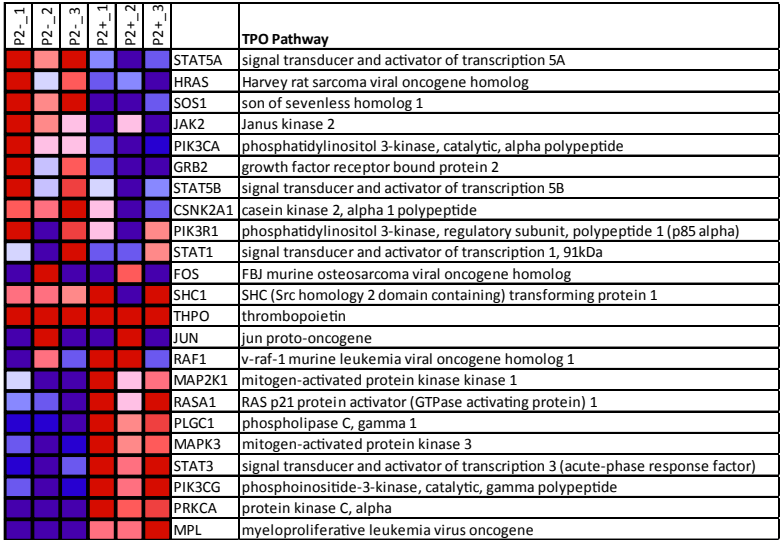

**D**

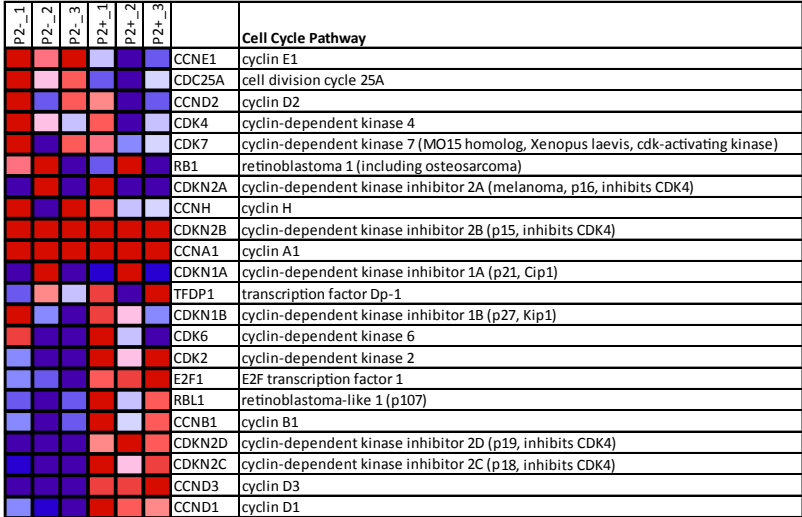

**E** P2+ Upregulated Diseases or Functions

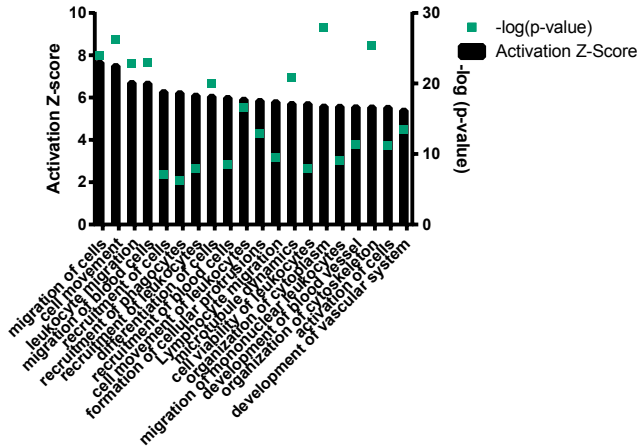

**C**

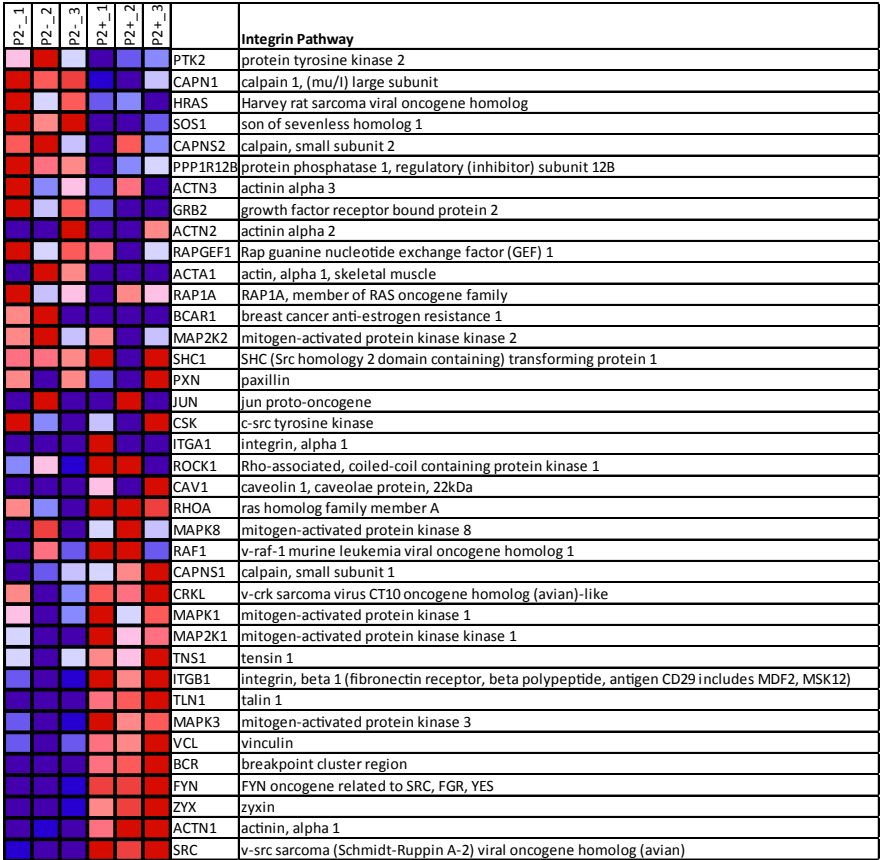

**F** P2+ Downregulated Diseases or Functions

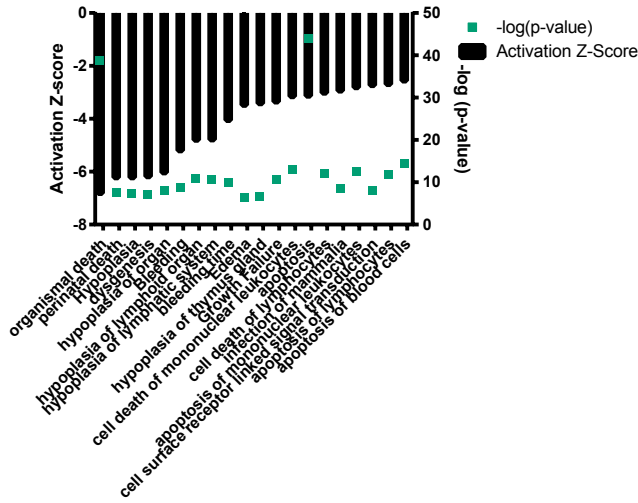

**G**

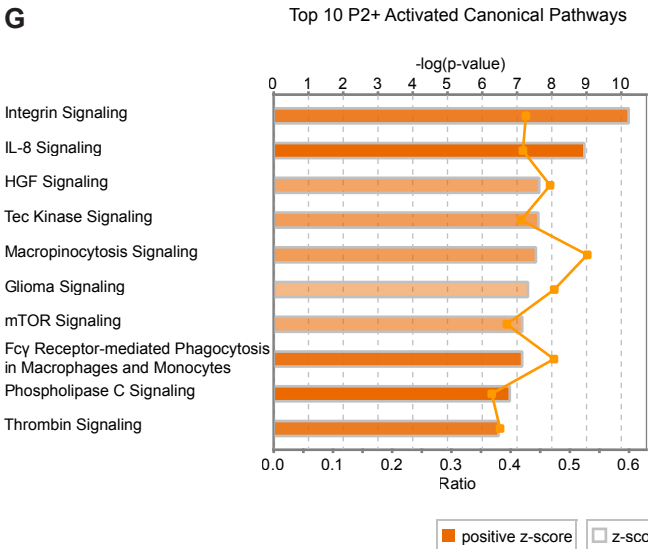

**H**

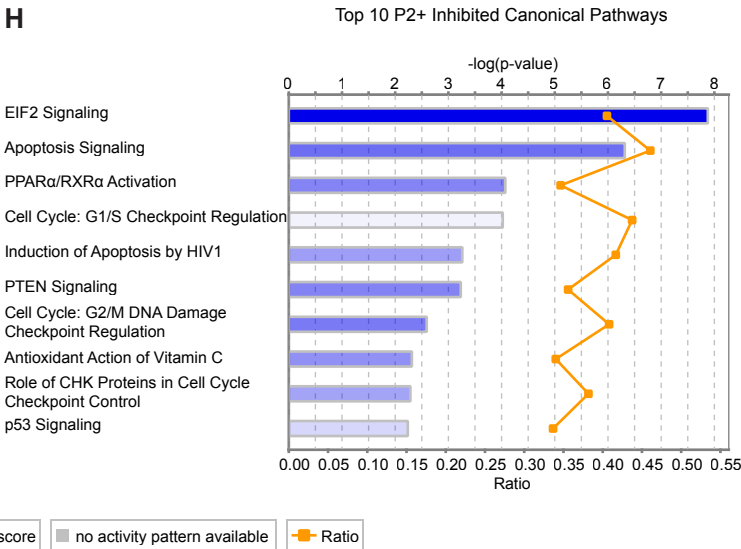

Supplement: S7 Fig — (Related to Fig 6) (A) Principal component analysis of the RNA Seq expression data from WT, P2- and P2+ PreMegE cells. (B-D) Heat maps depicting expression of gene sets from GSEA plots (displayed in Fig 6B) in P2- and P2+ PreMegE cells. (E–F) Diseases/functions upregulated (E) or downregulated (F) in P2+ PreMegEs compared to P2- PreMegEs as determined by IPA. (G–H) Signaling pathways activated (G) or inhibited (H) in P2+ PreMegEs compared to P2- PreMegEs as determined by IPA. (PDF) [file pgen.1005814.s007.pdf]

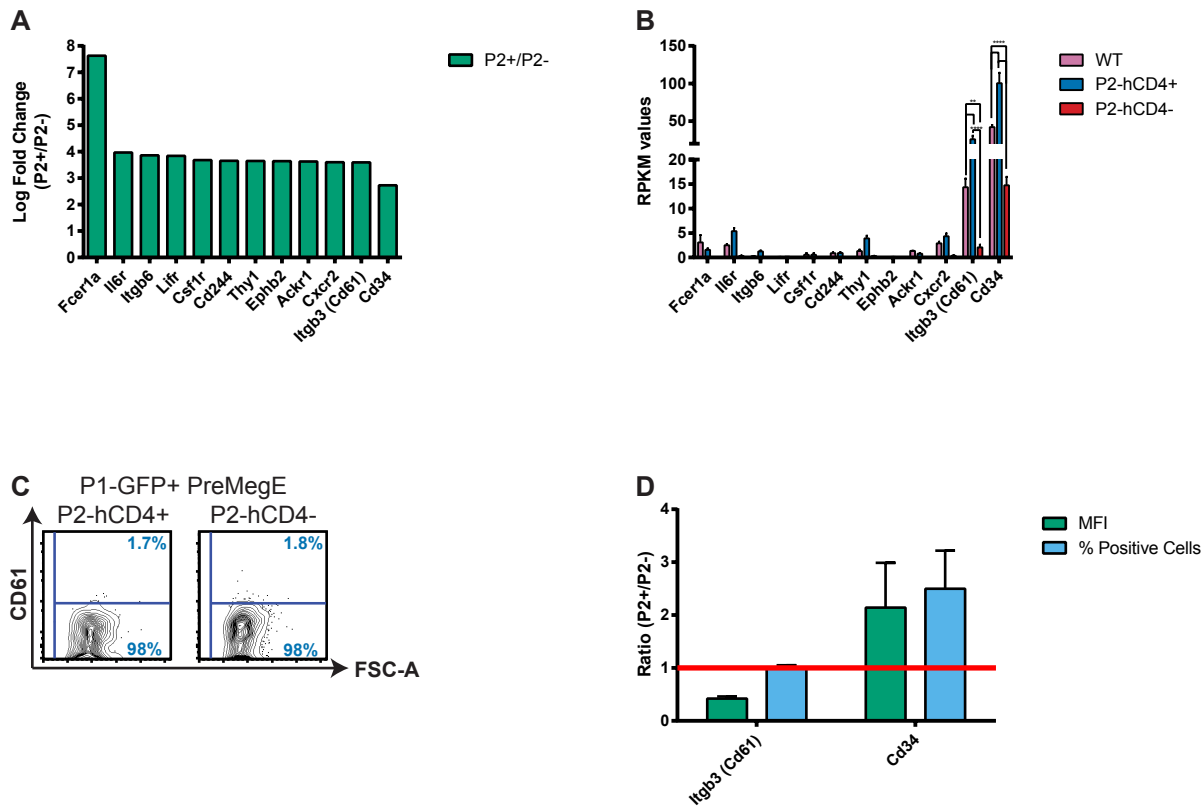

**E**

| WT PreMegE | No. cells per well | No. positive wells | % Mk only | % Ery only | % Mk + Ery |
|------------|--------------------|--------------------|-----------|------------|------------|
| CD34+      | 1                  | 44/144 (31%)       | 79.2      | 0          | 20.8       |
| CD34-      | 1                  | 30/144 (21%)       | 41.7      | 50         | 8.3        |

Supplement: S8 Fig — (Related to Fig 6) (A) Log fold change (counts) of cell surface markers in P2+ PreMegEs compared to P2- PreMegEs as determined by RNA Seq. (B) RPKM values of cell surface markers in WT, P2- and P2+ PreMegEs. (n = 3). (C) Representative FACS plot of CD61 expression in P2+ and P2- PreMegEs. (n = 2). (D) Ratio of median fluorescence intensity (MFI) and percentage of positive cells for CD61 and CD34 protein expression in P2+ PreMegEs compares to P2- PreMegEs, determined by FACS. (n = 2). (E) Table showing the clonal analysis of CD34+ and CD34- WT PreMegEs. Shown are numbers of positive wells at day 7 of OP9 co-culture relative to the numbers of single PreMegE cells plated on day 0 and the proportion of wells that contained CD41+ cells (Mk only), Ter119+ cells (Ery only) or CD41+ and Ter119+ cells (Mk + Ery). Data are compiled from 3 independent experiments. (PDF) [file pgen.1005814.s008.pdf]

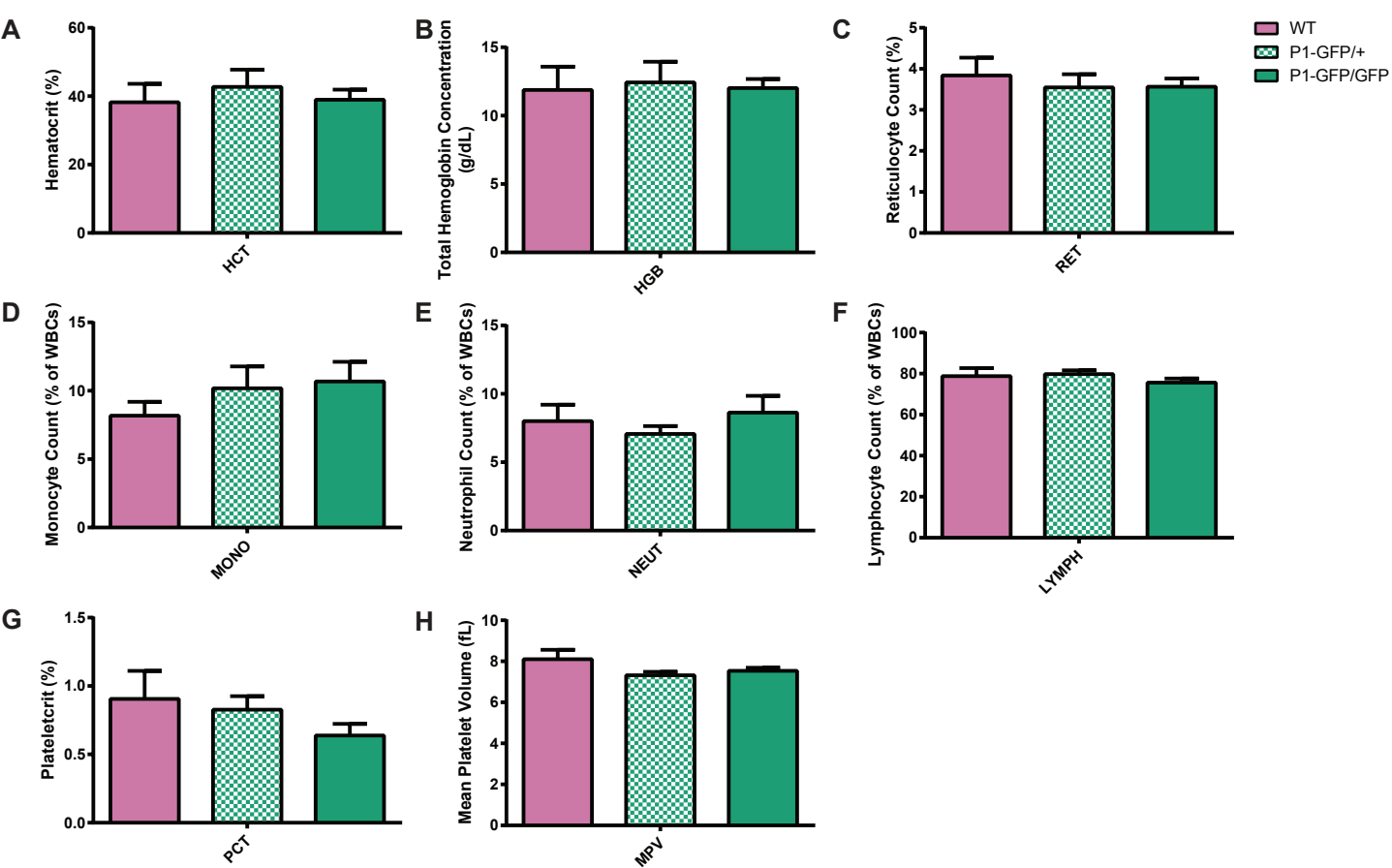

**S9 Fig.**

Supplement: S9 Fig — (Related to Fig 7) (A-H) Quantitation of Hematocrit (A), Total hemoglobin concentration (B), Reticulocyte count (C), Monocyte count (D), Neutrophil count (E), Lymphocyte count (F), Plateletcrit (G) and Mean Platelet Volume (H) in peripheral blood of WT, P1-GFP/+ and P1-GFP/GFP mice. (n = 4) (PDF) [file pgen.1005814.s009.pdf]

**A**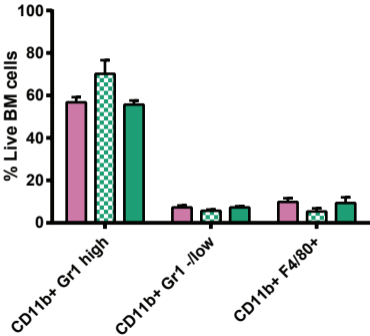**B**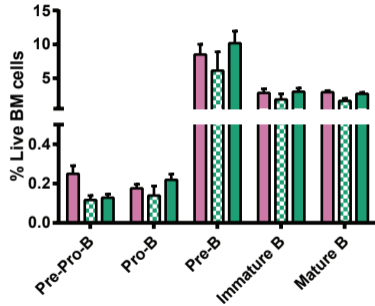**C**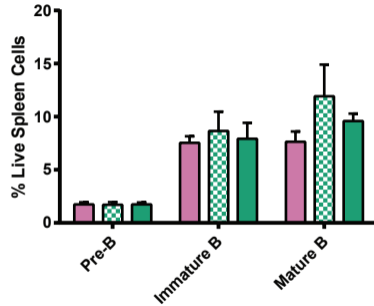

WT  
P1-GFP/+  
P1-GFP/GFP

**S10 Fig.**

Supplement: S10 Fig — (Related to Fig 7) (A—B) Numbers of GM (A) and B (B) lineage cells as a proportion of live ACK-lysed BM cells. (C) Numbers of B lineage cells as a proportion of live ACK-lysed spleen cells (n = 4). (PDF) [file pgen.1005814.s010.pdf]
