# Supplementary material for: RUNX1B Expression Is Highly Heterogeneous and Distinguishes Megakaryocytic and Erythroid Lineage Fate in Adult Mouse Hematopoiesis
Source: PLoS Genet. 2016 Jan 25;12(1):e1005814. doi: 10.1371/journal.pgen.1005814 (PMC4726605; doi:10.1371/journal.pgen.1005814)
Supplement: S3 Fig — (Related to Figs 3–6) (A-C) Gene expression analysis of total Runx1 (A), Runx1 P1 (B) and Runx1 P2 (C) in Lin- cKit+ HSPC populations isolated from WT BM, normalized to b-actin (n = 3). (PDF) [file pgen.1005814.s003.pdf]

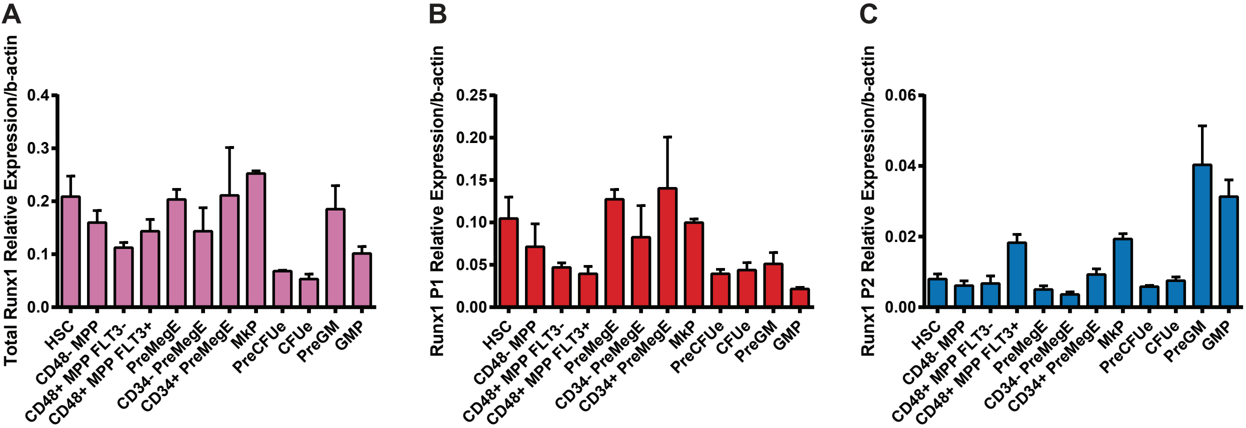
A
B
C
Total Runx1 Relative Expression/b-actin
Runx1 P1 Relative Expression/b-actin
Runx1 P2 Relative Expression/b-actin
0.0
0.1
0.2
0.3
0.4
0.00
0.05
0.10
0.15
0.20
0.25
0.00
0.02
0.04
0.06
HSC
CD48- MPP
CD48+ MPP FLT3-
CD48+ MPP FLT3+
PreMegE
CD34- PreMegE
CD34+ PreMegE
MkP
PreCFUe
CFUe
PreGM
GMP

S3 Fig.
