# Supplementary material for: RUNX1B Expression Is Highly Heterogeneous and Distinguishes Megakaryocytic and Erythroid Lineage Fate in Adult Mouse Hematopoiesis
Source: PLoS Genet. 2016 Jan 25;12(1):e1005814. doi: 10.1371/journal.pgen.1005814 (PMC4726605; doi:10.1371/journal.pgen.1005814)
Supplement: S1 Table — (DOCX) [file pgen.1005814.s012.docx]

**S1 Table. Details of Flow Cytometry Reagents**

| **Antibody** | **Conjugation** | **Clone** | **Reactivity** | **Host Species** | **Company** |
| --- | --- | --- | --- | --- | --- |
| CD3Ε | Biotin/APC/PE | 145-2C11 | Mouse | Armenian Hamster | eBioscience |
| CD4 | Biotin/eFluor 450 | GK1.5 | Mouse | Rat | eBioscience |
| CD4 | PE Alexa Fluor 610 | S3.5 | Human | Mouse | Invitrogen |
| CD4 | Alexa Fluor 700 | RPA-T4 | Human | Mouse | Biolegend |
| CD4 | APC/PE Cy7 | SK3 | Human | Mouse | eBioscience |
| CD5 | Biotin | 53-7.3 | Mouse | Rat | eBioscience |
| CD8A | Biotin/PerCPCy5.5 | 53-6.7 | Mouse | Rat | eBioscience |
| CD11B (MAC1) | Biotin/APC/  APC eFluor 780 | M1/70 | Mouse | Rat | eBioscience |
| CD16/CD32 (FCGRII/III) | Purified/Biotin/  PE Cy7/  Alexa Fluor 700/PE | 93 | Mouse | Rat | eBioscience |
| CD19 | APC eFluor 780/PE Cy7 | 1D3 | Mouse | Rat | eBioscience |
| CD25 | APC | PC61.5 | Mouse | Rat | eBioscience |
| CD27 (TNFRSF7) | Biotin | LG.7F9 | Human, Mouse, Rat | Armenian Hamster | eBioscience |
| CD34 | eFluor 660 | RAM34 | Mouse | Rat | eBioscience |
| CD41 | Biotin/APC/PE Cy7 | MWReg30 | Mouse | Rat | eBioscience |
| CD44 (PGP-1) | PE Cy7 | IM7 | Human, Mouse | Rat | eBioscience |
| CD45R (B220) | Biotin/PE/eFluor 450 | RA3-6B2 | Human,  Mouse | Rat | eBioscience |
| CD48 | APC | HM48-1 | Mouse | Armenian  Hamster | eBioscience |
| CD61 | APC | 2C9.G2 | Mouse, Rat | Armenian Hamster | Biolegend |
| CD71 (Transferrin Receptor) | Biotin | R17217 | Mouse | Rat | eBioscience |
| CD105 (Endoglin) | Biotin | MJ7/18 | Mouse | Rat | eBioscience |
| CD105 (Endoglin) | Pacific Blue | MJ7/18 | Mouse | Rat | Biolegend |
| CD117 (C-KIT) | APC eFluor 780 | 2B8 | Mouse, Pig | Rat | eBioscience |
| CD127 (IL7RA) | eFluor 450 | A7R34 | Mouse | Rat | eBioscience |
| CD135 (FLT3) | PE | A2F10 | Mouse | Rat | eBioscience |
| CD150 (SLAMF1) | Biotin/PE/PerCPCy5.5 | TC15-12F12.2 | Mouse | Rat | Biolegend |
| FCΕR1Α | Biotin/PE | MAR-1 | Mouse | Armenian Hamster | eBioscience |
| F4/80 | PE | BM8 | Mouse | Rat | eBioscience |
| IGD | Biotin | 11-26c | Mouse | Rat | eBioscience |
| IGM | APC | II/41 | Mouse | Rat | eBioscience |
| Integrin Beta 7 | PE | FIB504 | Human, Mouse | Rat | eBioscience |
| LY-6A/E (SCA1) | Biotin/Alexa Fluor 700/  PerCPCy5.5 | D7 | Mouse | Rat | eBioscience |
| LY-6C | eFluor 450 | HK1.4 | Mouse | Rat | eBioscience |
| LY-6G (GR1) | Biotin/PE Cy7 | RB6-8C5 | Mouse | Rat | eBioscience |
| TER119 | Biotin/PE | TER-119 | Mouse | Rat | eBioscience |
|  |  |  |  |  |  |
| **Reagent** | **Conjugation** | **Company** |  |  |  |
| Hoechst 33258 |  | Invitrogen |  |  |  |
| Streptavidin | PE Cy7/PerCP | eBioscience |  |  |  |
| Streptavidin | Pacific Orange | Invitrogen |  |  |  |
| 7-AAD |  | eBioscience |  |  |  |

7-AAD: 7-Aminoactinomycin D

APC: Allophycocyanin

Cy: Cyanine

PerCP: Peridinin Chlorophyll Protein
