# Supplementary material for: RUNX1B Expression Is Highly Heterogeneous and Distinguishes Megakaryocytic and Erythroid Lineage Fate in Adult Mouse Hematopoiesis
Source: PLoS Genet. 2016 Jan 25;12(1):e1005814. doi: 10.1371/journal.pgen.1005814 (PMC4726605; doi:10.1371/journal.pgen.1005814)
Supplement: S2 Table — (DOCX) [file pgen.1005814.s013.docx]

**S2 Table. Antibody combinations used for FACS analysis and sorting (see S1 table for clone and supplier details)**

| **Panel (refers to Figure no.)** | **Identified Populations** | **Primary Antibody/conjugate** | **Secondary Antibody/conjugate** |
| --- | --- | --- | --- |
| Total BM, spleen and thymus cells (Fig. 1B) | *Runx1* P1-GFP and P2-hCD4 expression in unfractionated BM, spleen and thymus | Anti-human CD4/PE Alexa Fluor 610 |  |
| BM Ery cells (Fig. 2A-B, 7E, S1A) | ProE  EryA  EryB  EryC | Anti-mouse CD71/Biotin  Anti-mouse Ter119/PE  Anti-human CD4/PE Alexa Fluor 610 | Streptavidin/PE Cy7 |
| BM GM cells (Fig. 2C-D, S1B, S10A) | CD11b+ Gr1 high  CD11b+ Gr1-/low  CD11b+ F4/80+ | Anti-mouse CD11B/APC  Anti-mouse Gr1/PE Cy7  Anti-mouse F4/80/PE  Anti-human CD4/PE Alexa Fluor 610 |  |
| BM and Spleen B cells (Fig. 2E-H, S1C-D, S10B-C) | Pre-Pro-B  Pro-B  Pre-B  Immature B  Mature B | Anti-mouse CD11B/PE  Anti-mouse CD3Ε/PE  Anti-mouse TER119/PE  Anti-mouse B220/eFluor 450  Anti-mouse C-KIT/APC eFluor 780  Anti-mouse CD19/PE Cy7  Anti-mouse IGM/APC  Anti-mouse IGD/biotin  Anti-human CD4/PE Alexa Fluor 610 | Streptavidin/Pacific Orange |
| Spleen and Thymus T cells (Fig. 2I-L, 7F-H, S1E-F) | DN1  DN2  DN3  DN4  CD4 CD8 DP  CD4 SP  CD8 SP | Anti-mouse CD4/eFluor 450  Anti-mouse CD8A/PerCPCy5.5  Anti-mouse CD44/PE Cy7  Anti-mouse CD25/APC  Anti-human CD4/PE Alexa Fluor 610 |  |
| BM LSK CD48- (Fig. 3A, C) | HSC  CD48- MPP | Lineage Cocktail: (anti-mouse CD3Ε/biotin, CD4/biotin, CD5/biotin, CD8A/biotin, B220/biotin, GR1/biotin, CD11B/biotin, TER119/biotin)  Anti-mouse CKIT/APC eFluor 780  Anti-mouse SCA1/PerCPCy5.5  Anti-mouse CD150/PE  Anti-mouse CD48/APC  Anti-human CD4/PE Cy7 | Streptavidin/Pacific Orange |
| BM LSK CD48+ (Fig. 3A, C) | CD48+ FLT3- MPP  CD48+ FLT3+ MPP | Lineage Cocktail  Anti-mouse CKIT/APC eFluor 780  Anti-mouse SCA1/PerCPCy5.5  Anti-mouse FLT3/PE  Anti-mouse CD48/APC  Anti-human CD4/PE Cy7 | Streptavidin/Pacific Orange |
| BM CLP (Fig. 3B-C) | CLP | Lineage Cocktail  Anti-mouse SCA1/PerCPCy5.5  Anti-mouse CD127/eFluor 450  Anti-mouse CKIT/APC eFluor 780  Anti-human CD4/APC | Streptavidin/Pacific Orange |
| B and T cell OP9/OP9-DL1 co-culture (Fig. 3D) | Cultured B220+ CD19+ and CD4 CD8 DP cells | Anti-mouse B220/PE  Anti-mouse CD19/PE Cy7  Anti-mouse CD4/eFluor 450  Anti-mouse CD8A/PerCPCy5.5 |  |
| Myeloid OP9 co-culture (Fig. 3G-H, 5I-J, 6G, S6C, S8E) | Cultured CD11B+ GR1+, CD41+ and TER119+ cells | Anti-mouse CD11b/APC eFluor 780  Anti-mouse GR1/PE Cy7  Anti-mouse CD41/APC  Anti-mouse TER119/PE |  |
| BM GM Progenitors (Fig. 4A-B) | GMP  PreGM | Lineage Cocktail  Anti-mouse SCA1/biotin  Anti-mouse CD41/biotin  Anti-mouse CKIT/APC eFluor 780  Anti-mouse CD150/PE  Anti-mouse CD16/32/PE Cy7  Anti-mouse Endoglin/Pacific Blue  Anti-human CD4/APC | Streptavidin/PerCP |
| BM MkE Progenitors (Fig. 5A-B) | PreMegE  MkP  PreCFUe  CFUe | Lineage Cocktail  Anti-mouse SCA1/biotin  Anti-mouse CD16/32/biotin  Anti-mouse CKIT/APC eFluor 780  Anti-mouse CD150/PE  Anti-mouse CD41/PE Cy7  Anti-mouse Endoglin/Pacific Blue  Anti-human CD4/APC | Streptavidin/Pacific Orange |
| BM-derived megakaryocytes (Fig. 5C) | CD41+ Mks | Anti-mouse CD41/PE Cy7  Anti-human CD4/APC |  |
| Cultured PreMegEs (Fig. 5K) | 12hr cultured PreMegEs | Lineage Cocktail  Anti-mouse SCA1/biotin  Anti-mouse CD16/32/biotin  Anti-mouse CKIT/APC eFluor 780  Anti-mouse CD150/PE  Anti-mouse CD41/PE Cy7  Anti-mouse Endoglin/Pacific Blue  Anti-human CD4/PE Alexa Fluor 610 | Streptavidin/ Pacific Orange |
| BM CD34 MkE Progenitors (Fig. 6E, S8D) | CD34+ PreMegE  CD34- PreMegE | Lineage Cocktail  Anti-mouse SCA1/biotin  Anti-mouse CD16/32/biotin  Anti-mouse CKIT/APC eFluor 780  Anti-mouse CD150/PE  Anti-mouse CD41/biotin  Anti-mouse Endoglin/Pacific Blue  Anti-mouse CD34/eFluor 660  Anti-human CD4/PE Cy7 | Streptavidin/ Pacific Orange |
| Peripheral Blood Multilineage (Fig. 7C) | Peripheral Blood CD3ε+, Mac1+ Gr1+, B220+ CD19+ | Anti-mouse CD3Ε/APC  Anti-mouse CD11B/PE  Anti-mouse GR1/PE Cy7  Anti-mouse B220/eFluor 450  Anti-mouse CD19/APC eFluor 780 |  |
| AML1-ETO9a-IRES-GFP BM HSPCs (Fig. 8) | AML1-ETO9a GFP+/- BM LSK, PreGM, GMP | Lineage Cocktail  Anti-mouse CKIT/APC eFluor 780  Anti-mouse SCA1/PerCPCy5.5  Anti-mouse CD150/PE  Anti-mouse CD41/APC  Anti-mouse CD16/32/PE Cy7  Anti-mouse Endoglin/Pacific Blue | Streptavidin/ Pacific Orange |
| Cultured LSK48F- MPPs – LSK FACS (S4 Fig.) | 18hr Cultured LSK48F- MPPs – LSK expression | Lineage Cocktail  Anti-mouse SCA1/Alexa Fluor 700  Anti-mouse cKit APC eFluor 780  Anti-human CD4 Alexa Fluor 610 | Streptavidin/ Pacific Orange |
| Cultured LSK48F- MPPs – LK MkE FACS (S4 Fig.) | 18hr Cultured LSK48F- MPPs – MkE marker expression | Lineage Cocktail  Anti-mouse SCA1/biotin  Anti-mouse CD16/32/Alexa Fluor 700  Anti-mouse CKIT/APC eFluor 780  Anti-mouse CD150/PE  Anti-mouse CD41/PE Cy7  Anti-mouse Endoglin/Pacific Blue  Anti-human CD4/PE Alexa Fluor 610 | Streptavidin/ Pacific Orange |
| Cultured GM Progenitors – mature GM cell markers (S5A-B Fig.) | Day 7-11 Cultured PreGM and GMP cells – GM markers | Anti-mouse F4/80/PE  Anti-mouse GR1/PE Cy7  Anti-mouse CD11B/APC |  |
| Cultured GM Progenitors – mast cell markers (S5A-B Fig.) | Day 7-11 Cultured PreGM and GMP cells – mast cell markers | Anti-mouse FCΕR1Α/PE  Anti-mouse CD11B/APC  Anti-mouse CKIT/APC eFluor 780 |  |
| BM Mast Cell Progenitor (S5C Fig.) | MCp | Lineage Cocktail  Anti-mouse SCA1/biotin  Anti-mouse FCΕR1Α/biotin  Anti-mouse CD27/biotin  Anti-mouse CKIT/APC eFluor 780  Anti-mouse Int Beta7/PE  Anti-mouse Ly6c/eFluor 450  Anti-human CD4/APC | Streptavidin/ PE Cy7 |
| Cultured GM Progenitors – Progenitor FACS (53D-E Fig.) | 12hr Cultured PreGM and GMP cells | Lineage Cocktail  Anti-mouse SCA1/biotin  Anti-mouse CD41/biotin  Anti-mouse CKIT/APC eFluor 780  Anti-mouse CD150/PE  Anti-mouse CD16/32/Alexa Fluor 700  Anti-mouse Endoglin/Pacific Blue  Anti-human CD4/PE Alexa Fluor 610 | Streptavidin/ Pacific Orange |
| PreMegE Cell Cycle (S6B Fig. | G0/G1, S and G2/M-phase PreMegEs | Lineage Cocktail  Anti-mouse SCA1/biotin  Anti-mouse Endoglin/biotin  Anti-mouse CD41/biotin  Anti-mouse CD16/32/biotin  Anti-mouse CD150/PerCPCy5.5  Anti-mouse CKIT/APC eFluor 780  Anti-human CD4/Alexa Fluor 700 | Streptavidin/ PE Cy7  (Click-iT EdU assay components: Alexa Fluor 647 Azide, FxCycle Violet) |
| BM CD61 MkE Progenitors (S8C-D Fig.) | CD61+ PreMegE  CD61- PreMegE | Lineage Cocktail  Anti-mouse SCA1/biotin  Anti-mouse CD16/32/biotin  Anti-mouse CKIT/APC eFluor 780  Anti-mouse CD150/PE  Anti-mouse CD41/biotin  Anti-mouse Endoglin/Pacific Blue  Anti-mouse CD61/APC  Anti-human CD4/PE Cy7 | Streptavidin/ Pacific Orange |

7-AAD: 7-Aminoactinomycin D

APC: Allophycocyanin

Cy: Cyanine

PerCP: Peridinin Chlorophyll Protein
