# Supplementary material for: RUNX1B Expression Is Highly Heterogeneous and Distinguishes Megakaryocytic and Erythroid Lineage Fate in Adult Mouse Hematopoiesis
Source: PLoS Genet. 2016 Jan 25;12(1):e1005814. doi: 10.1371/journal.pgen.1005814 (PMC4726605; doi:10.1371/journal.pgen.1005814)
Supplement: S3 Table — (DOCX) [file pgen.1005814.s014.docx]

**S3 Table. Primers and Probes used for qPCR**

| Target Gene | Primer 1 | Primer 2 | Roche Universal ProbeLibray Probe ID |
| --- | --- | --- | --- |
| Runx1 | ctccgtgctacccactcact | atgacggtgaccagagtgc | 77 |
| Runx1 P1 | gaagtgtaagcccagcacagt | ggcgggggattctataattt | 40 |
| Runx1 P2 | aagatccgagcccctgtc | tcacaacaagccgattgagt | 17 |
| Gata1 | ccctgaactcgtcataccact | gaacactggggttgaacctg | 83 |
| Gfi1b | tacccctgccagttctgtg | cttgtggggcttctcacct | 31 |
| Klf1 | caagagctcgcacctcaag | gagcgaacctccagtcaca | 68 |
| Epor | gtcctcatctcgctgttgct | atgccaggccagatcttct | 56 |
| Itga2b (Cd41) | tgctgctgaccctgctagt | gtcgattccgcttgaagaag | 97 |
| Itgb3 (Cd61) | gtgggagggcagtcctcta | caggatatcaggacccttgg | 31 |
| Mpl | tagctcccaaggcttcttcc | tcttcacatttctcccaggtg | 18 |
| Pf4 | catctcctctgggatccatct | ccattcttcagggtggctat | 9 |
| Cd34 | gggtagctctctgcctgatg | tccgtggtagcagaagtcaa | 84 |
| b-actin | tgacaggatgcagaagaaga | cgctcaggaggagcaatg | 106 |
| AML1-ETO9a | accactacagggactcctatcg | tcttgacgtgtgccatgc | 5 |
