# Supplementary material for: RUNX1B Expression Is Highly Heterogeneous and Distinguishes Megakaryocytic and Erythroid Lineage Fate in Adult Mouse Hematopoiesis
Source: PLoS Genet. 2016 Jan 25;12(1):e1005814. doi: 10.1371/journal.pgen.1005814 (PMC4726605; doi:10.1371/journal.pgen.1005814)
Supplement: S1 File — (DOCX) [file pgen.1005814.s016.docx]

**Supplemental Methods**

**Animal Husbandry and Tissue Collection**

*P1-GFP::P2-hCD4, P1-GFP*, *AML1-ETO9a-IRES-GFP/rtTA* and wild type littermate control mice were backcrossed with C57BL/6 mice for at least 10 generations and were housed in specific pathogen free cages with environmental enrichment. Spleen, thymus or bone (femurs and tibiae) tissues were collected from young adult mice (age 12-16 weeks, males and females) following humane culling by dislocation of the neck and confirmation of death according to Schedule 1 of the Animal Scientific Procedures Act (ASPA) 1986. Tissues were manually dissociated in DMEM supplemented with 10% FBS (Sigma-Aldrich) prior to fluorescence activated cell sorting and/or analysis; spleens and thymuses were crushed with a pestle and mortar and bones were flushed with a 23G needle plus 1ml syringe. Unless otherwise stated, a sample size of n = 1 refers to tissues collected from one adult mouse. Animals were identified according to breeding pair number, litter number and earsnip number and the genotype was recorded following sample preparation and identification by flow cytometry.

**RNA Sequencing**

Sequence reads were aligned to the mouse reference genome GRCm38/mm10 using Bowtie2 (version 2.2.1, default settings).  Gene annotation was taken from Ensembl release 76.  The expression levels of 41,228 annotated features were determined by using the featureCounts function from the Bioconductor package Rsubread (version 1.13.13).

The Bioconductor package edgeR (version 3.8.5) was used to identify genes that showed statistically significant variation in expressions level when comparing P2- and P2+ samples.  The data was filtered to include only genes with at least 1 count-per-million reads in at least 3 samples. The data were then normalised with the TMM (trimmed mean and M values) method and differential expression analysis was performed using the function exactTest in edgeR (>2-fold, false discovery rate <0.05). Gene set enrichment analysis (GSEA) software was used to perform against the BioCarta database (version 5, <http://www.biocarta.com/genes/index.asp>) [[1](#_ENREF_1),[2](#_ENREF_2)]. Data were also analysed using QIAGEN’s Ingenuity Pathway Analysis software (IPA, QIAGEN Redwood City, www.qiagen.com/ingenuity).

**Microscopy**

Methylcellulose colonies were photographed using a Leica DMI 3000 microscope and an N PLAN L lens (magnification 10, numerical aperture 0.25). Images were acquired using a Leica DFC 310 FX camera and Leica Application Suite Advanced Fluorescence Version 2.5.0.6735 acquisition software. MegaCult^TM^ culture slides were scanned using a Leica SCN400.

***In silico* assessment of transcription factor binding motifs**

Genomic DNA sequences from the mouse and human *Runx1/RUNX1*  loci were downloaded from the Mouse Dec. 2011 (GRCm38/mm10) and Human Dec. 2013 (GRCh38/hg38) Assemblies from the UCSC browser[[3](#_ENREF_3)] and submitted to rVISTA[[4](#_ENREF_4),[5](#_ENREF_5)] to review the presence of conserved EKLF, FLI1, GATA1 and TAL1 binding sites. The positions of the uploaded sequences are as follows:

*P1:* Human chromosome 21: 36,410,000-36,430,000; Mouse chromosome 16: 92,812,000-92,932,000

*P2:* Human chromosome 21: 36,250,000-36,270,000; Mouse chromosome 16: 92,690,000-92,710,000

ChIP-Seq data was downloaded from the Mouse ENCODE Consortium, having been generated by the Ross Hardison Lab, PennState[[6](#_ENREF_6),[7](#_ENREF_7)]. GEO series accession numbers: GSM923575, GSM923582, GSM923586, GSM995447. Data was viewed in the UCSC browser[[3](#_ENREF_3)].

**Supplemental References**

1. Subramanian A, Tamayo P, Mootha VK, Mukherjee S, Ebert BL, et al. (2005) Gene set enrichment analysis: a knowledge-based approach for interpreting genome-wide expression profiles. Proc Natl Acad Sci U S A 102: 15545-15550.

2. Mootha VK, Lindgren CM, Eriksson KF, Subramanian A, Sihag S, et al. (2003) PGC-1alpha-responsive genes involved in oxidative phosphorylation are coordinately downregulated in human diabetes. Nat Genet 34: 267-273.

3. Kent WJ, Sugnet CW, Furey TS, Roskin KM, Pringle TH, et al. (2002) The human genome browser at UCSC. Genome Res 12: 996-1006.

4. Frazer KA, Pachter L, Poliakov A, Rubin EM, Dubchak I (2004) VISTA: computational tools for comparative genomics. Nucleic Acids Res 32: W273-279.

5. Loots GG, Ovcharenko I, Pachter L, Dubchak I, Rubin EM (2002) rVista for comparative sequence-based discovery of functional transcription factor binding sites. Genome Res 12: 832-839.

6. Consortium EP (2012) An integrated encyclopedia of DNA elements in the human genome. Nature 489: 57-74.

7. Mouse EC, Stamatoyannopoulos JA, Snyder M, Hardison R, Ren B, et al. (2012) An encyclopedia of mouse DNA elements (Mouse ENCODE). Genome Biol 13: 418.
