# Supplementary material for: RUNX1B Expression Is Highly Heterogeneous and Distinguishes Megakaryocytic and Erythroid Lineage Fate in Adult Mouse Hematopoiesis
Source: PLoS Genet. 2016 Jan 25;12(1):e1005814. doi: 10.1371/journal.pgen.1005814 (PMC4726605; doi:10.1371/journal.pgen.1005814)
Supplement: S11 Fig — (A-B) rVISTA analysis of EKLF, FLI1, GATA1 and TAL1 binding motifs located in regions surrounding Runx1 P1 (A) and Runx1 P2 (B). Top, Blue: All motifs found in the mouse loci. Top, Green: Motifs conserved between mouse and human. Middle: Genetic conservation between mouse and human loci. Bottom: Schematic of mouse Runx1 locus, aligned to conservation map. (C) UCSC Browser tracks of mouse Runx1 locus, featuring GATA1 and TAL1 ChIP-seq data (performed in megakaryocytes and erythroblasts) acquired by the Mouse ENCODE Project, aligned to UCSC Runx1 transcripts and vertebrate genetic conservation data. (PDF) [file pgen.1005814.s011.pdf]

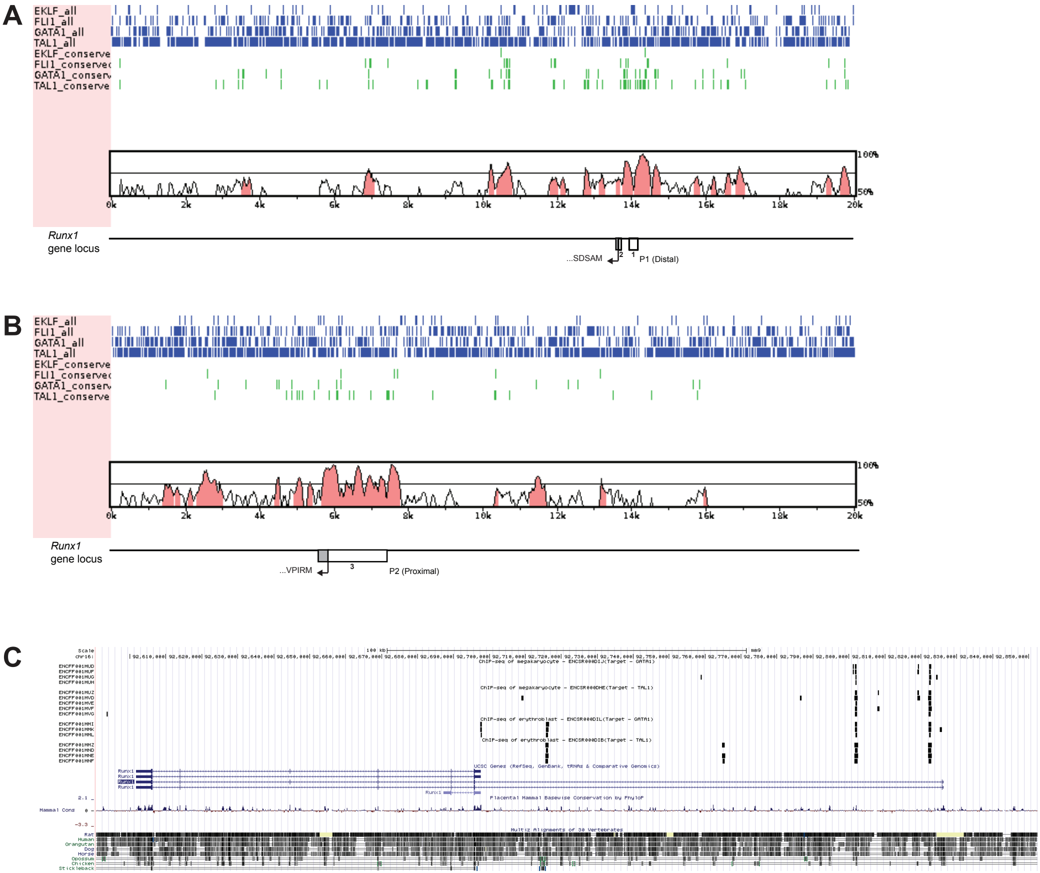
A
EKLF_all
FLI1_all
GATA1_all
TAL1_all
EKLF_conserve
FLI1_conserved
GATA1_conserv
TAL1_conserve
100%
50%
0k
2k
4k
6k
8k
10k
12k
14k
16k
18k
20k
Runx1
gene locus
2
1
...SDSAM
P1 (Distal)
B
EKLF_all
FLI1_all
GATA1_all
TAL1_all
EKLF_conserve
FLI1_conserved
GATA1_conserv
TAL1_conserve
100%
50%
0k
2k
4k
6k
8k
10k
12k
14k
16k
18k
20k
Runx1
gene locus
3
...VPIRM
P2 (Proximal)
C
Scale
chr16:
100 kb
mm9
ChIP-seq of megakaryocyte - ENCSR000DIJ(Target - GATA1)
ChIP-seq of megakaryocyte - ENCSR000DHE(Target - TAL1)
ChIP-seq of erythroblast - ENCSR000DIL(Target - GATA1)
ChIP-seq of erythroblast - ENCSR000DIB(Target - TAL1)
UCSC Genes (RefSeq, GenBank, tRNAs & Comparative Genomics)
Runx1
Placental Mammal Basewise Conservation by PhyloP
Mammal Cons
Multiz Alignments of 30 Vertebrates
Rat
Human
Orangutan
Dog
Horse
Opossum
Chicken
Stickleback

S11 Fig.
